# Genetic Variability of the Functional Domains of Chromodomains Helicase DNA-Binding (CHD) Proteins

**DOI:** 10.3390/genes12111827

**Published:** 2021-11-19

**Authors:** Ana R. Cardoso, Mónica Lopes-Marques, Manuela Oliveira, António Amorim, Maria J. Prata, Luísa Azevedo

**Affiliations:** 1 i3S—Instituto de Investigação e Inovação em Saúde, Universidade do Porto, Rua Alfredo Allen 208, 4200-135 Porto, Portugal; acardoso@ipatimup.pt (A.R.C.); mmarques@i3s.up.pt (M.L.-M.); manuelao@ipatimup.pt (M.O.); aamorim@ipatimup.pt (A.A.); mprata@ipatimup.pt (M.J.P.); 2 IPATIMUP—Institute of Molecular Pathology and Immunology, University of Porto, Rua Júlio Amaral de Carvalho 45, 4200-135 Porto, Portugal; 3 FCUP—Department of Biology, Faculty of Sciences, University of Porto, Rua do Campo Alegre, s/n, 4169-007 Porto, Portugal

**Keywords:** chromodomains helicase DNA-binding protein, neurodevelopment, chromatin remodelling, transcription regulation, evolutionary conservation

## Abstract

In the past few years, there has been an increasing neuroscientific interest in understanding the function of mammalian chromodomains helicase DNA-binding (CHD) proteins due to their association with severe developmental syndromes. Mammalian CHDs include nine members (CHD1 to CHD9), grouped into subfamilies according to the presence of specific functional domains, generally highly conserved in evolutionary terms. Mutations affecting these domains hold great potential to disrupt protein function, leading to meaningful pathogenic scenarios, such as embryonic defects incompatible with life. Here, we analysed the evolution of CHD proteins by performing a comparative study of the functional domains of CHD proteins between orthologous and paralogous protein sequences. Our findings show that the highest degree of inter-species conservation was observed at Group II (CHD3, CHD4, and CHD5) and that most of the pathological variations documented in humans involve amino acid residues that are conserved not only between species but also between paralogs. The parallel analysis of both orthologous and paralogous proteins, in cases where gene duplications have occurred, provided extra information showing patterns of flexibility as well as interchangeability between amino acid positions. This added complexity needs to be considered when the impact of novel mutations is assessed in terms of evolutionary conservation.

## 1. Introduction

Mammalian chromodomains helicase DNA-binding (CHDs) proteins represent a family of nine proteins involved in chromatin remodelling and transcription regulation during mammalian development [1,2,3,4,5,6,7,8,9]. All these proteins present two chromodomains and two helicase domains and yet other specific functional domains permit to distinguish three groups of CHDs: I (CHD1 and CHD2), II (CHD3, CHD4, and CHD5), and III (CHD6, CHD7, CHD8, and CHD9) [1]. Group II proteins possess two additional plant homeodomains (PHD), responsible for the physical binding of CHDs to the Nucleosome Remodelling Deacetylase (NuRD) chromatin-remodelling complex, which plays a critical role in embryonic development [10,11,12]. Group III proteins present a Brahma and Kismet (BRK) domain with unclear functions [1,8,13]. Additionally, both group I and III proteins have smaller domains such as the SWI3, ADA2, N-COR, and TFIIB (SANT), whereas group I and II CHDs retain domains of unknown significance (DUF) [1,8,13].

Regardless of the mammalian model used, the central roles of CHDs in embryonic viability and in utero development have been firmly demonstrated either by in vitro or in vivo studies. As such, CHD1 affects cell proliferation and growth during mouse embryonic development [14]; CHD2 is associated with murine neurogenesis and memory formation [3,15]; CHD3 is essential for brain development [16]; CHD4 orchestrates neuronal connectivity and embryonic vascular development [17,18]; CHD5 regulates murine spermiogenesis [19]; CHD6 is associated with mouse coordination and ataxia [20]; CHD7 is involved in mice cell proliferation and neuronal differentiation [21]; CHD8 is connected to murine neurodevelopment and autistic-like features [22]; and CHD9 is involved in mouse oocyte chromatin remodelling, possibly being associated with epigenetic reprogramming [23]. In humans, defective CHDs have been linked to severe phenotypes such as Pilarowski-Bjornsson Syndrome [24], Epileptic Encephalopathy, childhood-onset [25,26], Snijders Blok-Campeau Syndrome [27], Sifrim-Hitz-Weiss Syndrome [28,29], CHARGE Syndrome [30,31,32,33,34] and autism spectrum disorder [35,36,37,38,39]. Noteworthy, a significant proportion of mutations associated with to these pathologies were mapped into conserved functional domains [24,27,28,31,34,35,40,41,42].

Given the impact of CHDs in human disease, in vivo studies using model organisms are crucial to obtain insights into the cascade of pathophysiological events that leads to the disorder. Lin and colleagues [43] reported several differences between mice and human transcriptional landscapes, partially explained by the metabolic and physiological differences among the species. If murine CHDs have been the focus of most studies performed until now [2,3,16,17,19,20,21,44,45,46,47], the near prospects are to extend the strategy to other non-human mammals because they are assumed to be the most suitable models for brain research. Evolutionary constraints are crucial for understanding the history of these proteins and to predict the impact that new mutations will eventually have on the protein. However, the current knowledge on the degree of similarity in structure and sequence between protein members of the CHDs family in those model organisms is still very limited.

As a better understanding of the genetic diversity associated with the functional domains of CHDs is essential for future research on neurodevelopmental and neurological disorders, we performed a comparative analysis of the functional domains of CHD proteins between *Homo sapiens* and five non-human mammals by compilingCHD pathogenic mutations to investigate their degree of conservation in orthologous as well as in paralogous sequences.

## 2. Materials and Methods

### 2.1. Sequence Retrieval and Alignment of CHD Proteins

Human canonical transcripts for each nine CHD protein were retrieved from the UniProt database [48]. Six mammalian species were selected—human (*Homo sapiens*), chimpanzee (*Pan troglodytes*), Rhesus macaque (*Macaca mulatta*), domestic cat (*Felis catus*), rat (*Rattus norvegicus*), and mouse (*Mus musculus*). Orthologous sequences were searched in the HomoloGene database from National Centre for Biotechnology Information (NCBI) (https://www.ncbi.nlm.nih.gov/homologene. Accessed on 2 November 2020) [49]. In cases where more than one transcript existed for the target protein, Position-Specific Iterated BLAST (PSI-BLAST) algorithm (https://blast.ncbi.nlm.nih.gov/Blast.cgi. Accessed on 2 November 2020) [50] was used to determine the highest homology transcript to each canonical human transcript retrieved from UniProt [48]. The reference for each sequence used in this work is displayed in Table S1. Additionally, we retrieved three protein sequences of the invertebrate *Caenorhabditis elegans* (UniProt: O61845, Q22516, and O17909) referred in the work of Flanagan and colleagues [51]. A global alignment including all nine sets of CHD proteins and their orthologues (54 sequences) was performed using the Clustal Omega tool (Max HMM Iterations = 5) (https://www.ebi.ac.uk/Tools/msa/clustalo/. Accessed on 2 November 2020) [52]. The resulting alignment file was refined using the default options of BMGE software [53] available at (https://ngphylogeny.fr/tools/. Accessed on 2 November 2020) [54,55] (BLOSUM 62 Matrix, Maximum Entropy Threshold = 0.5, Gap Rate Cut-off = 0.5 and Minimum Block Size = 5).

### 2.2. Phylogenetic Analysis of CHD Proteins

The refined sequence alignment file from BMGE was used to calculate a phylogenetic tree using the built it SMS [56] option in PhyML 3.0 (http://www.atgc-montpellier.fr/phyml/. Accessed on 9 July 2021) [57] resulting in JTT+G+I. Posterior probabilities were determined using aBayes [58]. The tree analysis was performed until the average standard deviation of split frequencies approximated to 0. The final phylogenetic tree was then midpoint rooted and edited with the online software iTOL (https://itol.embl.de/shared_projects.cgi. Accessed on 9 July 2021) [59].

### 2.3. Alignment of CHD Functional Domains

We identified the amino acid sequences for each functional domain of human CHD proteins from the UniProt database (Chromo 1, Chromo 2, Helicase ATP-binding, Helicase C-terminal, PHD I, and PHD II) [48]. The amino acid sequences of SANT, DUF, and BRK domains were retrieved from the Conserved Domain Database (CDD) from NCBI (https://www.ncbi.nlm.nih.gov/cdd/. Accessed on 16 March 2021) [60] and Simple Modular Architecture Research Tool (SMART) (http://smart.embl-heidelberg.de. Accessed on 16 March 2021) [61]. Sizes and coordinates of each domain were retrieved from the aforementioned databases. We used Clustal Omega (https://www.ebi.ac.uk/Tools/msa/clustalo. Accessed on 16 March 2021) [52] to compare the functional domains between: (a) *H. sapiens* and the five orthologue species *P. troglodytes*, *M. mulatta*, *F. catus*, *R. norvegicus*, and *M. musculus* (interspecific analysis); and (b) Paralogues of the same group (I, II, III) in *H. sapiens* (intraspecific analysis). MView (https://www.ebi.ac.uk/Tools/msa/mview. Accessed on 2 November 2020) [62] was used to analyze the results.

### 2.4. Retrieval of Pathogenic Missense Variants

On 2 March 2021, we retrieved the pathogenic missense variants affecting CHDs domains from the ClinVar database (https://www.ncbi.nlm.nih.gov/clinvar. Accessed on 2 March 2021) [63]. The filters used for our query were (1) Clinical significance—pathogenic and (2) Molecular consequence—missense. We only considered pathogenic variants previously reported in the literature. The pathogenic missense variants were located in the alignments of the functional domains.

## 3. Results

### 3.1. Phylogenetic Analysis of CHD Proteins

**Figure 1:**
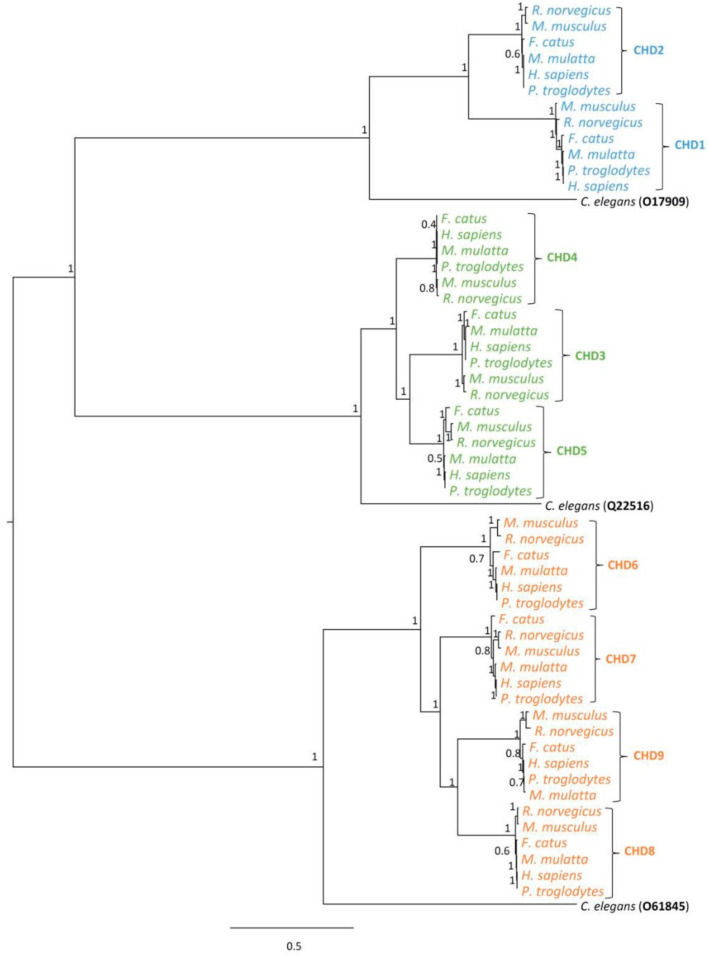
Phylogenetic tree for the CHD family of proteins. Evolutionary model was determined automatically using the built it SMS [56] option in PhyML 3.0 [57] resulting in JTT+G+I. Branch support values shown at tree nodes correspond to posterior probabilities determined using aBayes [58]. The highlighting represents each protein group (blue: group I; green: group II and orange: group III).

The phylogenetic analysis of CHD proteins considering the six mammalian species under investigation (Figure 1) showed that CHDs cluster in three clades that match the three groups of proteins from the CHD family based on the types of functional domains: I (CHD1 and CHD2), II (CHD3, CHD4, and CHD5), and III (CHD6, CHD7, CHD8, and CHD9). According to Flanagan and colleagues [51], each group of CHDs derived from one of three different ancestral proteins. In line with this and taking into account the position of the sequences from *C. elegans*, our analysis suggests that at least three ancestral CHDs existed before the divergence of vertebrates [6,51], an indication that the expansion of the CHD gene family by gene duplication events occurred after the divergence between invertebrates and vertebrates. Flanagan and colleagues [51] further showed that invertebrates, vertebrates and plants have three CHD classes whereas some unicellular organisms have one CHD group. As such, the question can be raised on whether the proliferation of different paralogues for CHD proteins might have occurred in the transition from unicellular to multicellular organisms due to the demanding needs of specialized functions that emerged with multicellularity [64,65,66]. The phylogenetic topology for group III CHDs shows that CHD6 places basally to CHD7, CHD8, and CHD9, indicating that the CHD6 sequence is more divergent than CHD7, CHD8, or CHD9. This result is consistent with those provided by the biochemical studies of Manning and Yusufzai [7] which suggest that CHD6 lacks nucleosome sliding activity and might have a different mechanism to disrupt chromatin, in contrast to other group III CHDs.

### 3.2. Analysis of Domains between Human CHDs

**Figure 2:**
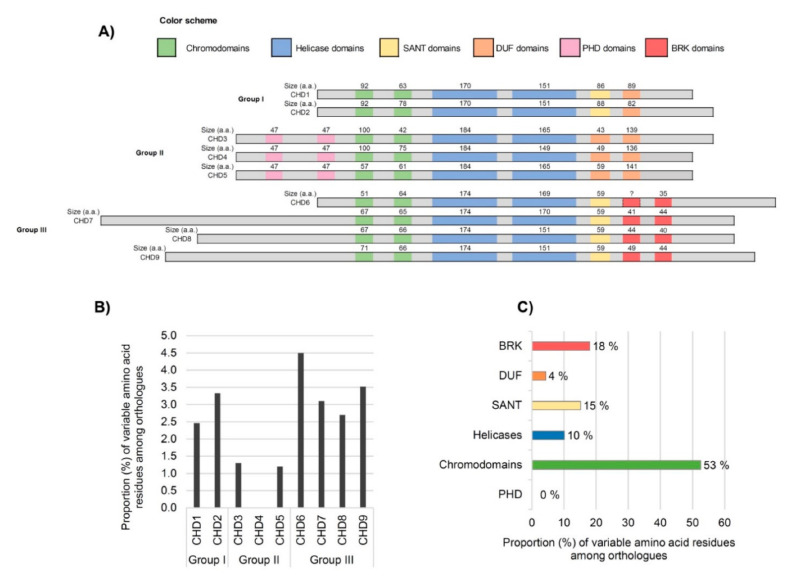
Schematic representation of the architecture of CHD proteins. (**A**) Amino acid (a.a.) size of the conserved domains for each group of CHD paralogues in *Homo sapiens*. (**B**) Percentage of variable amino acid residues within the functional domains of CHD proteins among orthologues distributed by groups I, II, and III (*n* = 139). (**C**) Proportion of variable amino acid residues within the functional domains of CHD proteins among orthologues distributed by categories—Chromodomains, Helicases, SWI3, ADA2, N-COR, and TFIIB (SANT), Domains of Unknown Significance (DUF), Brahma and Kismet (BRK), and Plant Homeodomains (PHD) (*n* = 139).

Next, to dissect the domain architecture of human CHDs, we performed alignments of the amino acid sequences (Figure 2A). The number of amino acids of the shared domains was similar among members of the CHD family group, with only a few exceptions, such as the Chromo 1 domain between Group II proteins (Figure 2A). The largest Chromo 1 domain is present in Group II (CHD3 and CHD4) and the smallest in Group III (CHD6). This domain shows high intra- and inter-group heterogeneity in terms of segment size. The helicase domain of CHDs, consisting of two modules: one Helicase ATP-binding and one Helicase C-terminal, is of extreme importance because it provides energy for nucleosome remodeling through its ATPase activity [13,27]. The Helicase ATP-binding has remained invariable in terms of amino acid content between all CHD proteins belonging to the same group, whereas the other helicase domain (C-terminal domain) reveals heterogeneity not only between groups but also considerably among members of groups II and III. In what concerns the group-specific domains DUF and BRK, our comparative analyses revealed some differences among members of the same group.

Regarding the BRK domain(s) of the CHD6 protein, there is still contradictory information on whether it contains one BRK domain or two tandem BRK domains, as is reflected in the published schematic representations of the human CHD family [1,7,8,13]. For this reason, the first CHD6′s BRK domain is signalized with a question mark in Figure 2A (for sequence details see Figure S1) showing the conserved residues. For the second BRK domain, the SMART analysis retrieved a BRK domain of 35 a.a. in CHD6, as is represented in Figure 2A. The SANT domain of group III CHDs is shorter than group I. However, the size is the same in all four members (59 a.a.) and members of group I only differ by 2 amino acids; reinforcing the importance of this domain. The PHD domains, specific to group II CHDs, are well conserved in terms of size.

### 3.3. Inter-Species Comparison of CHD Domains

The comparative analysis of the functional domains’ sequences between humans and non-human primates, revealed 100% identity among H. sapiens, P. troglodytes, and M. mulatta (Figure S2), indicating that CHD domains are extremely conserved in primates. Concerning the comparison between humans and the other non-primate mammalian species (*F. catus*, *R. norvegicus*, and *M. musculus*), a total of 139 variable amino acid positions were detected within the Chromodomains, Helicases, SANT, DUF, BRK, and PHD of all nine CHDs (Figure 2B,C). The highest percentage of variable residues (4.5%) is observed on CHD6, whereas CHD4 displays no variability within functional domains between the species analyzed. The lowest levels of inter-species heterogeneity were observed at Group II (CHD3, CHD4, and CHD5). Since all members of this group are important components of the NuRD complex [10], the observed high level of conservation of this group might be an indication that the maintenance of the domain structure of the group is important to preserve the binding affinities to the NuRD complex, contributing to its functionality and allowing it to orchestrate the biological processes in which is involved. Besides, there are lines of evidence that CHD4 also plays fundamental NuRD-independent functions, including the proliferation of neural precursors and regulation of neuronal connectivity, whereas CHD3 and CHD5 have been associated with neuron differentiation and migration [17,18,67]. The degree of conservation of Group II CHDs domain among mammalian species, clearly indicates the relevance of the biological pathways, many still unknown, in which these CHDs participate.

The highest average proportion of inter-species heterogeneity was found among the members of group III. One of these proteins is CHD7 which was associated with CHARGE syndrome, the most well-established syndrome connected with the CHD family of proteins. Different mouse models were already reported for this syndrome exhibiting phenotypes that mimic human symptoms [68]. According to our data, 3.3% of the CHD7 domain sequence is variable among the orthologues from *F. catus*, *R. norvegicus*, and *M. musculus.* CHD9 also shows a high number of variable residues among orthologues (Figure 2B). Although CHD9 continues to be the less well-characterized member of the subfamily III [47], its role in the regulation of expression of osteogenic cells [69,70] has been well documented. In contrast, a recent study revealed that mice with depleted CHD9 survived and were fertile, indicating that CHD9 is dispensable for murine embryonic development [47]. The same study demonstrated that acute depletion of CHD9 in human cancer cells elicited more robust gene expression changes, suggesting that CHD9 is a highly context-dependent chromatin regulator. Thus, further studies are needed to elucidate if the amino acid differences between human and murine CHD9 influence the functions of this protein and its associated pathological phenotypes. Additionally worth mentioning, the proportion of amino acid differences among orthologues peaked in CHD6 (4.5%), a finding that suggests that the selective constraints related to this protein are more relaxed. Fittingly, mouse lines with deletion of part of the ATPase domain of CHD6 were reported to be viable [20].

The distribution of amino acid differences by domain category (Figure 2C) revealed that most of the variations fall within the chromodomains category (53%) whereas the PHD domains do not accommodate any variation in the six species of mammals herein considered. In eukaryotes, there are various protein families containing chromodomains, which are modules 40–100 amino acids long that despite being evolutionarily conserved, are diversified enough to allow their classification into different groups [51,71]. While the most well-known function of chromodomains is to recognize and bind to lysine-methylated histone tails, facilitating recruitment to chromatin [72,73], other important functions have been proposed, such as direct nucleic acid recognition and binding [72,73].

The analysis of the structure of chromodomains revealed that the most conserved region is limited to 21 residues that form a conserved core in each chromodomain, referred to as a chromobox, which has motifs differing between the double chromodomains as well as between modules of the nine CHD proteins [51]. Chromoboxes harbour critical aromatic residues for methyllysine recognition, namely at position 5 where there is always an aromatic residue, although other aromatic residues at positions 8 and/or 12 are required for methyllysine binding capability of chromodomains [74]. The observation that the chromodomains accumulate the highest proportion of amino acid differences among orthologues is rather puzzling, but meaningfully most of the differences detected were outside the chromoboxes (Figure S2), and among those inside a chromobox, none involved positions 5, 8 or 12.

CHD7 presented the highest proportion of variable residues at chromoboxes, with four positions varying among orthologues located there: p.Ile843Val (*M. mulatta*, *F. catus*, *R. norvegicus* and *M. musculus*), p.Arg920Leu (all non-primates), p.Arg921Lys (*M. musculus*), p.Ile924Leu (*R. norvegicus*). Comparatively to the chromodomains, the helicase domains present a much lower proportion of variable amino acid residues among orthologues (10%) despite having on average twice or more the size of the chromodomains. Many pathogenic mutations implicated in neurodevelopmental disorders were reported to cluster on these domains whose disruption activates pathogenic mechanisms associated with ATP hydrolysis [13,27,35].

The extreme conservation of the PHDs is quite interesting. These domains are uniquely present in the group II CHDs, where they are featured in two tandem modules that share very high sequence homology in the three human proteins. PHDs are central “readers” of histone post-translational modifications that control gene expression cascades by recruiting multiprotein complexes consisting of chromatin regulators and transcription factors. The majority of the PHDs characterized to date bind to unmodified or methylated states of histone H3 lysine 4 [75,76]. Different subclasses of PHD fingers are present in distinct PHD-containing proteins (over 100 human proteins containing this module), in which mutations disrupting PHD fingers have been associated with a wide range of human diseases, including immunological disorders, neurological syndromes (e.g., Sotos syndrome, Rubenstein-Taybi Syndrome, etc.), and cancer [75,77].

### 3.4. Analyses of the Pathological Diversity Associated with CHDs

Overall, although our previous data revealed that the six different domains harboured by CHDs are in general highly conserved among humans and the mammalian species here addressed, some changes seem to be tolerated, particularly concerning the chromodomains. This opened the challenge of further dissecting how our findings could provide insights into the genetic basis of CHD’s associated neurodevelopmental disorders. So, we extended the analysis towards mapping the described CHDs pathogenic variants in the context of the level of conservation of the different domains either among paralogues or orthologues. Viewing that, we used the NIH open-access database ClinVar to obtain the data set of CHDs pathogenic variants implying one of the six domains, being of note that all retrieved variants were classified as de novo mutations in the platform. This is not surprising since it is well documented that de novo mutations, including germinal and postzygotic mutations, are important players in the genetic architecture of neurodevelopmental disorders [78], and specifically involving CHDs de novo mutations in CHD8 have been recurrently found in autism [36,38].

**Figure 3:**
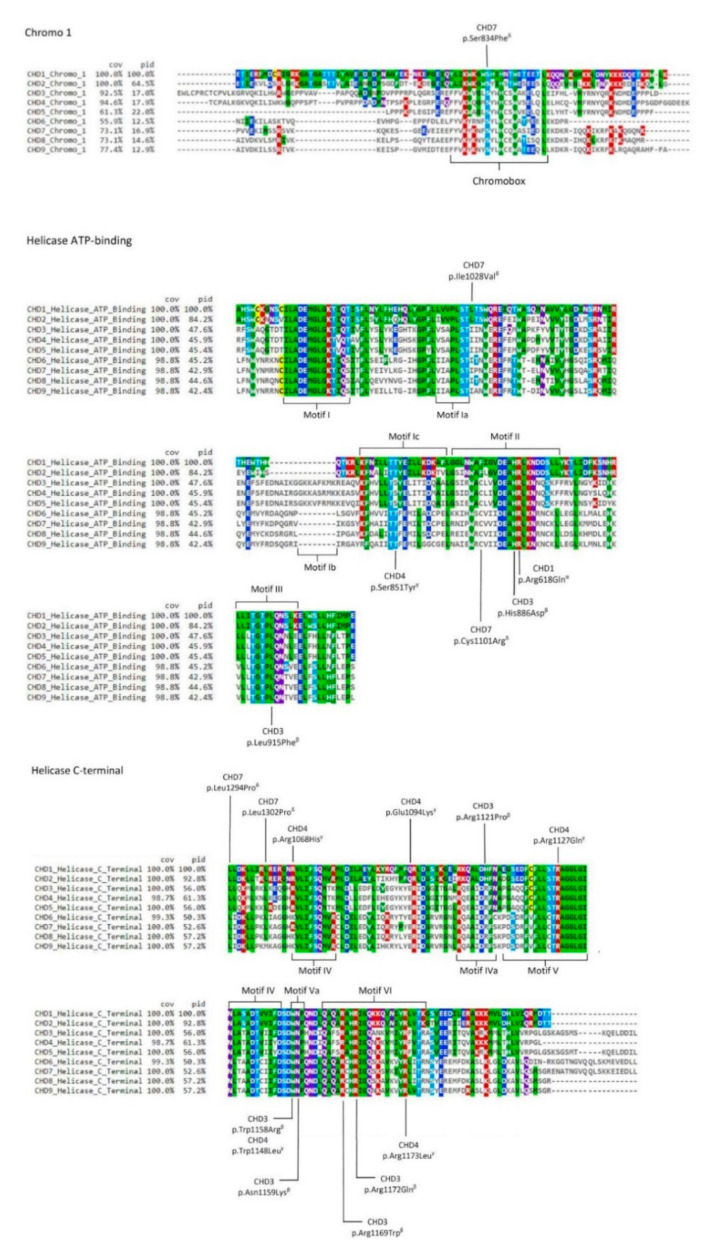
Scheme of CHD paralogues with pathogenic variants obtained from the ClinVar database (https://www.ncbi.nlm.nih.gov/clinvar. Accessed on 2 November 2020) [63] also described in the literature (Table 1) for domains Chromo 1, Helicase ATP-binding, and Helicase C-terminal. Letters α, β, γ, and δ represent the clinical phenotypes associated with each pathogenic variant (see Table 1). Chromobox and motifs I, Ia, Ib, Ic, II, III, IV, IVa, V, Va, and VI are displayed. The colour scheme represents the variable amino acid positions after using the multiple alignment viewer MView (COV—% coverage; ID—% identity). The mapping of the chromobox and the motifs was performed according to references [27,28,51,85,86].

The filtered CHDs variants are present in Table 1 whereas Figure 3 displays their location in the specific human sequences that are aligned with the paralogue regions. The overwhelming majority of these pathogenic variants are located at one of the two tandem helicase domains.

The helicase domains of the superfamily 2 of helicases, to which CHDs belong, have 11 signature motifs (I, Ia, Ib, Ic, II, III, IV, IVa, V, Va, and VI, marked in boxes in Figure 3) crucial for protein activity [79]. Almost all mutations we are now addressing, cluster within or near one of these highly conserved motifs, especially motifs V and VI of the Helicase C-terminal, and motifs Ia, II and III of Helicase ATP-binding. The two mutations affecting the VI motif are located in the highly conserved arginine fingers.

The mutations residing in the Helicase C-terminal involve highly conserved residues among mammalian orthologues that, in general, are also invariable paralogue sites (seven in a total of ten mutations). One exception was p.Arg1121Pro in CHD3, which according to Snijders Blok and colleagues [27] changes a residue in a helix region that is predicted to affect the structure of the CHD3 protein and as demonstrated through functional studies to lower enzymatic activity. In human CHD1 and CHD2, the corresponding position is occupied by histidine (Figure S1), which is, like arginine, a positively charged amino acid, demonstrating the residue flexibility among paralogues. Contrarily, the pathological effects of the substitution of an arginine by a proline are quite predictable, given the very distinct properties of the two amino acids.

As a counterexample, we can take p.Trp1158Arg, again in CHD3, a variant that was previously shown to affect the chromatin remodelling ability of the enzyme [27]. In this case, the alteration involved a position where tryptophan was found to be highly conserved among paralogues, which suggests that the substitution of the residue in any of the paralogue proteins can be critical. Fittingly, Weiss and colleagues [80] performed a functional study on the variant p.Trp1148Leu in CHD4 that revealed its role in decreasing chromatin remodelling activity, a result rather similar to that reported for p.Trp1158Arg in CHD3. The obvious inference is that mutations affecting highly conserved positions among paralogue polypeptide segments will likely disturb the protein function of any of the paralogues. Snijders Blok and colleagues [27] also provided evidence that the arginines located at positions 1169 and 1172 of CHD3 are crucial for correct enzymatic function, further establishing that p.Arg1172Gln caused reduced ATPase activity in functional studies. We cannot omit to highlight that these two positions are highly conserved among orthologues and paralogues (Table 1 and Figure 3), reinforcing their importance for the function of all CHDs.

The pathogenic mutations in the Helicase ATP-binding domain also locate predominately in the highly conserved motifs of the core region (Figure 3). Whilst half of the mutations involved invariable residues in all paralogues, the remaining half are conserved only among paralogues belonging to the same group of CHDs. Among the latter is, for instance, the p.Cys1101Arg in CHD7, found in CHARGE patients [82]. Cysteine is present in all the members of group II and III CHDs (CHD3 to CHD9), but instead, CHD1 and CHD2 have phenylalanine in the homologous position. At this same residue of CHD7 Bergman and colleagues [82] identified in a patient also with CHARGE syndrome a tyrosine (p.Cys1101Tyr), an amino acid with similar properties to phenylalanine. Thus, cysteine at this position in CHD7 seems to be highly intolerant to change, leading to infer a crucial importance for the protein function and probably also for all members from group II and III (CHD3 to CHD9).

Out of the examined CHDs pathogenic mutations, only one changed the sequence of a chromodomain, which was p.Ser834Phe in CHD7, specifically located within the Chromo 1 domain. As mentioned, in each chromodomains of all CHDs there is a chromobox that represents the highly conserved core of the module [51]. It consists of 21-residues among which Ser834 that was here observed to be invariant in the comparison between orthologues or paralogues (Figure 3), further supporting the importance of this particular amino acid residue for the correct function of the chromodomains. In CHD7 this residue lies just next to one of the two aromatic amino acids that form an aromatic cage in chromodomains [73]. Since the aromatic cages of the chromodomains are thought to play a crucial role in the coordination of the recognition of histone methylated-lysine [73], it seems likely that the substitution p.Ser834Phe, given that it introduces an additional aromatic residue, might interfere with the aromatic cage itself, disrupting its function.

## 4. Discussion

The present work aimed to enrich the knowledge on CHD proteins, a family of enzymes associated with severe developmental disorders. The investigation of sequence variability residing in the evolutionarily conserved domains of the CHD family of proteins allowed us to consolidate and extend the current knowledge on these crucial proteins, highlighting some features that previously had been scarcely documented or addressed namely in what concerns the patterns of conservation of amino acid residues between orthologous sequences.

Firstly, the family of CHD proteins is of very ancient origin and the diversification of this protein family appears to have accompanied the increased requirements of multicellularity, bearing out the essentiality of a set of proteins known to be regulators of transcription and critical players during developmental processes.

Secondly, the mapping of deleterious variants in distinct CHDs, revealed they predominantly cluster in non-variable positions either among the nine human paralogues or among paralogues belonging to the same group of CHDs. Furthermore, most of the mutated positions laid in highly conserved motifs within specific catalytic domains, meaning that the disruption of those sequences results in severe disease phenotypes. In this analysis, we have only focused on CHD mutations causing Mendelian or quasi-mendelian neurodevelopmental disorders. Due to that, the search in the ClinVar only yielded results for missense mutations in CHD1, CHD2, CHD3, CHD4, and CHD7. However, a plethora of pathogenic mutations in these and other members of CHD proteins has been identified in more complex neurodevelopmental and psychiatric diseases and even in cancer. For instance, mutations in CHD8 have often been found in ASD patients [22,35,36,39], and very recently data provided by An and colleagues [35] indicated that mutations affecting the helicase domains of that protein were associated with more severe ASD phenotypes. CHD8 has also recently been implicated in Zahir Friedman syndrome (ZFS) [87]. The gene encoding CHD5 was also found to be a shared risk locus in ASD and two psychiatric disorders [88], fitting the observation that the effects of CHD5 deletion in mouse were consistent with the presentation of ASDs [89].

CHDs remodelers are also potent tumour suppressors and their disruption contribute to the development of a variety of cancers [90,91]. Among the members implicated in cancer, CHD6 is an illustrative example, as it was recently demonstrated to be a key regulator of the oxidative DNA damage response in a manner importantly dependent on the roles exerted by the double chromodomains and SANT domain of CHD6 [90]. A mutation at the CHD6 second SANT domain was identified in a patient clinically presenting Hallermann-Streiff syndrome, a rare premature aging disorder [92]. In a mouse model it was shown that depletion of part of the ATPase domain of CHD6 interferes with motor coordination, indicating that CHD6 could be responsible for some ataxia phenotypes [20].

The case of CHD6 illustrates not only how deficiencies in individual CHD proteins can lead to dissimilar diseases but also how disparate phenotypes can result from mutations in distinct domains or even in the same domain. The complexity of CHDs functions in is still far from being fully understood. Still, as Manning and Yusufzai [7] suggested, the huge diversity of dys/malfunctions associated with CHD paralogues may rely on their non-redundant remodelling activities, on the expression control of specific paralogs in a tissue/temporal dependent fashion, and on the interactions between the paralogs with different sets of sequence-specific transcription factors.

Taken together, all these observations indicate that the variability present among the catalytic domains of paralogues is important to the specialization of functions distributed by the different proteins. At the same time, it may also be noted that the conservation observed between paralogues in key amino acid residues implies that protein function is the result of a network of fine intra-molecular interactions [93,94,95] which can be dramatically impaired by a point alteration at any of these critical sites resulting in pathological conditions. Overall we show that the comparative analysis of both orthologous and paralogous proteins reveals patterns of tolerability and flexibility that represent valuable insights for an accurate prediction of the impact of mutations.

## Figures and Tables

**Table 1:** Collection of the described human deleterious mutations (de novo) affecting conserved domains of CHD proteins. Data obtained from the ClinVar database (https://www.ncbi.nlm.nih.gov/clinvar. Accessed on 2 November 2020) [63] and also described in the literature.

Protein	Mutation	Domain	Phenotype	Inheritance	Orthologue Variability	Paralogue Variability	References
CHD1	p.Arg618Gln	Helicase ATP-binding (493-663 a.a.)	Pilarowski-Bjornsson Syndrome (α)	Autosomal Dominant	No	No	Pilarowski et al. (2018) [24]
CHD3	p.Leu915Phe p.His886Asp	Helicase ATP-binding (748–932 a.a.)	Snijders Blok-Campeau Syndrome, Intellectual disability (β)	Autosomal Dominant	No	No	Snijders Blok et al. (2018) [27]
	p.Arg1121Pro, p.Trp1158Arg p.Asn1159Lys p.Arg1169Trp p.Arg1172Gln	Helicase C-terminal (1064–1229 a.a.)	Snijders Blok-Campeau Syndrome, Intellectual disability (β)	Autosomal Dominant	No	Yes (p.Arg1121Pro)	Snijders Blok et al. (2018) [27]
CHD4	p.Ser851Tyr	Helicase ATP-binding (738–922 a.a.)	Sifrim-Hitz-Weiss syndrome (γ)	Autosomal Dominant	No	Yes	Sifrim et al. (2016) [29]
	p.Arg1068His p.Glu1094Lys p.Arg1127Gln p.Trp1148Leu p.Arg1173Leu	Helicase C-terminal (1054–1203 a.a.)	Sifrim-Hitz-Weiss syndrome (γ)	Autosomal Dominant	No	Yes (p.Arg1068His and p.Glu1094Lys)	Sifrim et al. (2016) [29] Weiss et al. (2016) [28] Weiss et al. (2020) [80] Richards et al. (2015) [81]
CHD7	p.Ser834Phe	Chromo 1 (800–867 a.a.)	CHARGE association, Idiopathic hypogonadotropic hypogonadism (δ)	Autosomal Dominant	No	No	Delahaye et al. (2007) [33] Kim et al. (2008) [41]
	p.Ile1028Val p.Cys1101Arg	Helicase ATP-binding (980-1154 a.a.)	CHARGE association (δ)	Autosomal Dominant	No	Yes	Vissers et al. (2004) [32] Bergman et al. (2011) [82]
	p.Leu1294Pro p.Leu1302Pro	Helicase C-terminal (1294–1464 a.a.)	CHARGE association (δ)	Autosomal Dominant	No	Yes (p.Leu1302Pro)	Hale et al. (2016) [83] Lalani et al. (2006) [84] Legendre et al. (2017) [42]

## Data Availability

The data presented in this study are available in this paper and supplementary material.

## Supplementary Materials

The following are available online at https://www.mdpi.com/article/10.3390/genes12111827/s1, Figure S1: Multiple sequence alignment scheme of the first Brahma and Kismet (BRK) domain among group III chromodomains helicase-DNA binding (CHD) protein orthologues (CHD6, CHD7, CHD8 and CHD9), Figure S2: Multiple sequence alignment of chromodomains helicase-DNA binding (CHD) protein orthologues (Hsap: *Homo sapiens*; Ptro: *Pan troglodytes*; Mmul: *Macaca mulatta*; Fcat: *Felis catus*; Rnor: *Rattus norvegicus*; Mmus: *Mus musculus*), Table S1: Accession numbers of CHD protein sequences used in the phylogenetic tree.
