# Supplementary material for: Genetic Variability of the Functional Domains of Chromodomains Helicase DNA-Binding (CHD) Proteins
Source: Genes (Basel). 2021 Nov 19;12(11):1827. doi: 10.3390/genes12111827 (PMC8623811; doi:10.3390/genes12111827)
Supplement: Supplementary file 1 [file genes-12-01827-s001.zip › genes-1413632-supplementary.pdf]

Supplementary materials

Genetic variability of the functional domains of Chromodomains Helicase DNA-Binding (CHD) proteins

Ana R. Cardoso[1,2,3], Mónica Lopes-Marques[1,2,3], Manuela Oliveira[1,2,3], António Amorim[1,2,3], Maria João Prata[1,2,3], Luísa Azevedo[1,2,3]

[1] i3s- Instituto de Investigação e Inovação em Saúde, Universidade do Porto, Population Genetics and Evolution Group, Rua Alfredo Allen 208, 4200-135 Porto, Portugal

[2] IPATIMUP-Institute of Molecular Pathology and Immunology, University of Porto, Rua Júlio Amaral de Carvalho 45, 4200-135 Porto, Portugal

[3] Department of Biology, Faculty of Sciences, University of Porto, Rua do Campo Alegre, s/n, 4169-007 Porto, Portugal

Table S1- Accession numbers of CHD protein sequences used in the phylogenetic tree. The human canonical transcripts were retrieved from UniProt [48].

Protein	*Homo sapiens*	*Pan troglodytes*	*Macaca mulatta*	*Felis catus*	*Rattus norvegicus*	*Mus musculus*
**CHD1**	NP_001261.2	XP_517850.3	XP_001097125.1	XP_023114504.1	NP_001100935.1	NP_031716.2
**CHD2**	NP_001262.3	XP_003952781.2	XP_028707159.1	XP_011281040.2	NP_001100993.1	NP_001074814.2
**CHD3**	NP_001005273.1	XP_003339343.1	NP_001361419.1	XP_023099697.1	XP_017453283.1	NP_666131.3
**CHD4**	NP_001264.2	XP_016778332.1	XP_028685032.1	XP_023112488.1	XP_006237463.1	XP_006505349.1
**CHD5**	NP_056372.1	XP_016808514.1	XP_014996210.2	XP_023113778.1	NP_001258155.1	NP_001074845.1
**CHD6**	NP_115597.3	XP_016793413.1	XP_015004487.1	XP_019682057.1	XP_008760567.1	NP_775544.2
**CHD7**	NP_060250.2	XP_016815001.2	XP_015000905.1	XP_023104204.1	NP_001101376.2	NP_001264078.1
**CHD8**	NP_001164100.1	XP_003314292.2	XP_028707218.1	XP_019688333.1	NP_001334590.1	NP_963999.2
**CHD9**	NP_001295248.1	XP_016785317.1	XP_014981612.2	XP_023100872.1	XP_002725429.2	NP_001297459.1

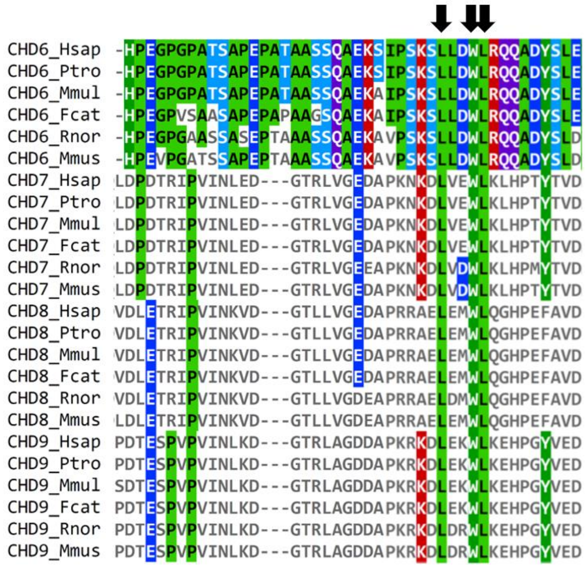

Figure S1- Multiple sequence alignment scheme of the first Brahma and Kismet (BRK) domain among group III chromodomains helicase-DNA binding (CHD) protein orthologues (CHD6, CHD7, CHD8 and CHD9). The coloring scheme represents the variable amino acid positions after using the multiple alignment viewer MView [62]. The black arrows represent the conserved positions among the sequences of this domain.

GROUP I

Chromo 1 (CHD1)
```
Hsap_CHD1 ETIERFMDCRIGRKGATGATTTIYAVEADGDPNAGFEKNKEPGEIQYLIKWKGWSHIHNTWETEETLKQQNVRGMKKLDNYKKKDQETKRWLK
Ptro_CHD1 ETIERFMDCRIGRKGATGATTTIYAVEADGDPNAGFEKNKEPGEIQYLIKWKGWSHIHNTWETEETLKQQNVRGMKKLDNYKKKDQETKRWLK
Mmul_CHD1 ETIERFMDCRIGRKGATGATTTIYAVEADGDPNAGFEKNKEPGEIQYLIKWKGWSHIHNTWETEETLKQQNVRGMKKLDNYKKKDQETKRWLK
Fcat_CHD1 ETIERFMDCRIGRKGATGATTTIYAVEADGDPNAGFEKNKEPGEIQYLIKWKGWSHIHNTWETEETLKQQNVRGMKKLDNYKKKDQETKRWLK
Rnor_CHD1 ETIERFMDCRVGRKGATGATTTIYAIEADGDPNAGFEKSKEPGDVQYLIKWKGWSHIHNTWETEETLKQQNVRGMKKLDNYKKKDQETKRWLK
Mmus_CHD1 ETIERVMDCRVGRKGATGATTTIYAVEADGDPNAGFERNKEPGDIQYLIKWKGWSHIHNTWETEETLKQQNVRGMKKLDNYKKKDQETKRWLK
```

Chromo 2 (CHD1)
```
Hsap_CHD1 QIVERIIAHSNQKSAAGYPDYYCKWQGLPYSECSWEDGALISKKFQACIDEYFSRNQSKTTPFK
Ptro_CHD1 QIVERIIAHSNQKSAAGYPDYYCKWQGLPYSECSWEDGALISKKFQACIDEYFSRNQSKTTPFK
Mmul_CHD1 QIVERIIAHSNQKSAAGYPDYYCKWQGLPYSECSWEDGALISKKFQACIDEYFSRNQSKTTPFK
Fcat_CHD1 QIVERIIAHSNQKSAAGYPDYYCKWQGLPYSECSWEDGALISKKFQACIDEYFSRNQSKTTPFK
Rnor_CHD1 QIVERIIAHSNQKSAAGLPDYYCKWQGLPYSECSWEDGALISKKFQTCIDEYFSRNQSKTTPFK
Mmus_CHD1 QIVERIIAHSNQKSAAGLPDYYCKWQGLPYSECSWEDGALISKKFQTCIDEYFSRNQSKTTPFK
```

Helicase ATP-Binding (CHD1)
```
Hsap_CHD1 AHSWCKGNSCILADEMGLGKTIQTISFLNYLFHEHQLYGPFLLVVPLSTLTSWQREIQTWASQMNAVVYLGDINSRNMIRTHEWTHHQTKRLK
Ptro_CHD1 AHSWCKGNSCILADEMGLGKTIQTISFLNYLFHEHQLYGPFLLVVPLSTLTSWQREIQTWASQMNAVVYLGDINSRNMIRTHEWTHHQTKRLK
Mmul_CHD1 AHSWCKGNSCILADEMGLGKTIQTISFLNYLFHEHQLYGPFLLVVPLSTLTSWQREIQTWASQMNAVVYLGDINSRNMIRTHEWTHHQTKRLK
Fcat_CHD1 AHSWCKGNSCILADEMGLGKTIQTISFLNYLFHEHQLYGPFLLVVPLSTLTSWQREIQTWASQMNAVVYLGDINSRNMIRTHEWMHPQTKRLK
Rnor_CHD1 AHSWCKGNSCILADEMGLGKTIQTISFLNYLFHEHQLYGPFLLVVPLSTLTSWQREIQTWASQMNAVVYLGDINSRNMIRTHEWMHPQTKRLK
Mmus_CHD1 AHSWCKGNSCILADEMGLGKTIQTISFLNYLFHEHQLYGPFLLVVPLSTLTSWQREIQTWASQMNAVVYLGDINSRNMIRTHEWMHPQTKRLK

Hsap_CHD1 FNILLTTYEILLKDKAFLGGLNWAFIGVDEAHRLKNDDSLLYKTLIDFKSNHRLLITGTPLQNSLKELWSLLHFIMPE
Ptro_CHD1 FNILLTTYEILLKDKAFLGGLNWAFIGVDEAHRLKNDDSLLYKTLIDFKSNHRLLITGTPLQNSLKELWSLLHFIMPE
Mmul_CHD1 FNILLTTYEILLKDKAFLGGLNWAFIGVDEAHRLKNDDSLLYKTLIDFKSNHRLLITGTPLQNSLKELWSLLHFIMPE
Fcat_CHD1 FNILLTTYEILLKDKAFLGGLNWAFIGVDEAHRLKNDDSLLYKTLIDFKSNHRLLITGTPLQNSLKELWSLLHFIMPE
Rnor_CHD1 FNILLTTYEILLKDKAFLGGLNWAFIGVDEAHRLKNDDSLLYKTLIDFKSNHRLLITGTPLQNSLKELWSLLHFIMPE
Mmus_CHD1 FNILLTTYEILLKDKAFLGGLNWAFIGVDEAHRLKNDDSLLYKTLIDFKSNHRLLITGTPLQNSLKELWSLLHFIMPE
```

Helicase C-Terminal (CHD1)
```
Hsap_CHD1 LLDKLLIRLRERGNRVLIFSQMVRMLDILAEYLKYRQFPFQRLDGSIKGELRKQALDHFNAEGSEDFCFLLSTRAGGLGINLASADTVVIFDS
Ptro_CHD1 LLDKLLIRLRERGNRVLIFSQMVRMLDILAEYLKYRQFPFQRLDGSIKGELRKQALDHFNAEGSEDFCFLLSTRAGGLGINLASADTVVIFDS
Mmul_CHD1 LLDKLLIRLRERGNRVLIFSQMVRMLDILAEYLKYRQFPFQRLDGSIKGELRKQALDHFNAEGSEDFCFLLSTRAGGLGINLASADTVVIFDS
Fcat_CHD1 LLDKLLIRLRERGNRVLIFSQMVRMLDILAEYLKYRQFPFQRLDGSIKGELRKQALDHFNAEGSEDFCFLLSTRAGGLGINLASADTVVIFDS
Rnor_CHD1 LLDKLLIRLRERGNRVLIFSQMVRMLDILAEYLKYRQFPFQRLDGSIKGELRRQALDHFNAEGSEDFCFLLSTRAGGLGINLASADTVVIFDS
Mmus_CHD1 LLDKLLIRLRERGNRVLIFSQMVRMLDILAEYLKYRQFPFQRLDGSIKGELRKQALDHFNAEGSEDFCFLLSTRAGGLGINLASADTVVIFDS

Hsap_CHD1 DWNPQNDLQAQARAHRIGQKKQVNIYRLVTKGSVEEDILERAKKKMVLDHLVIQRMDTT
Ptro_CHD1 DWNPQNDLQAQARAHRIGQKKQVNIYRLVTKGSVEEDILERAKKKMVLDHLVIQRMDTT
Mmul_CHD1 DWNPQNDLQAQARAHRIGQKKQVNIYRLVTKGSVEEDILERAKKKMVLDHLVIQRMDTT
Fcat_CHD1 DWNPQNDLQAQARAHRIGQKKQVNIYRLVTKGSVEEDILERAKKKMVLDHLVIQRMDTT
Rnor_CHD1 DWNPQNDLQAQARAHRIGQKKQVNIYRLVTKGSVEEDILERAKKKMVLDHLVIQRMDTT
Mmus_CHD1 DWNPQNDLQAQARAHRIGQKKQVNIYRLVTKGSVEEDILERAKKKMVLDHLVIQRMDTT
```

SANT (CHD1)
```
CHD1_Hsap KGFSDAEIRRFIKSYKKFGGPLERLDAIARDAELVDKSETDLRRLGELVHNGCIKALKDSSSGTERTGGRLGKVKGPTFRISGVQVN
CHD1_Ptro KGFSDAEIRRFIKSYKKFGGPLERLDAIARDAELVDKSETDLRRLGELVHNGCIKALKDSSSGTERTGGRLGKVKGPTFRISGVQVN
CHD1_Mmul KGFSDAEIRRFIKSYKKFGGPLERLDAIARDAELVDKSETDLRRLGELVHNGCIKALKDSSSGTERTGGRLGKVKGPTFRISGVQVN
CHD1_Fcat KGFSDAEIRRFIKSYKKFGGPLERLDAIARDAELVDKSETDLRRLGELVHNGCIKALKDNSSGTERTGGRLGKVKGPTFRISGVQVN
CHD1_Rnor KGFSDAEIRRFIKSYKKFGGPLERLDAIARDAELVDKSETDLRRLGELVHNGCVKALKDSSSGTERAGGRLGKVKGPTFRISGVQVN
CHD1_Mmus KGFSDAEIRRFIKSYKKFGGPLERLDAIARDAELVDKSETDLRRLGELVHNGCVKALKDSSSGTERAGGRLGKVKGPTFRISGVQVN
```

DUF4208 (CHD1)
```
CHD1_Hsap LDQKTFSICKERMRPVKAALKQLDRPEKGLSEREQLEHTRQCLIKIGDHITECLKEYTNPEQIKQWRKNLWIFVSKFTEFDARKLHKLYK
CHD1_Ptro LDQKTFSICKERMRPVKAALKQLDRPEKGLSEREQLEHTRQCLIKIGDHITECLKEYTNPEQIKQWRKNLWIFVSKFTEFDARKLHKLYK
CHD1_Mmul LDQKTFSICKERMRPVKAALKQLDRPEKGLSEREQLEHTRQCLIKIGDHITECLKEYTNPEQIKQWRKNLWIFVSKFTEFDARKLHKLYK
CHD1_Fcat LDQKTFSICKERMRPVKAALKQLDRPEKGLSEREQLEHTRQCLIKIGDHITECLKEYTNPEQIKQWRKNLWIFVSKFTEFDARKLHKLYK
CHD1_Rnor LDQKTFSICKERMRPVKAALKQLDRPEKGLSEREQLEHTRQCLIKIGDHITECLKEYSNPEQIKQWRKNLWIFVSKFTEFDARKLHKLYK
CHD1_Mmus LDQKTFSICKERMRPVKAALKQLDRPEKGLSEREQLEHTRQCLIKIGDHITECLKEYSNPEQIKQWRKNLWIFVSKFTEFDARKLHKLYK
```

Chromo 1 (CHD2)
```
Hsap_CHD2 ETIEKVLDSRLGKKGATGASTTVYAIEANGDPSGDFDTEKDEGEIQYLIKWKGWSYIHSTWESEESLQQQKVKGLKKLENFKKKEDEIKQWLG
Ptro_CHD2 ETIEKVLDSRLGKKGATGASTTVYAIEANGDPSGDFDTEKDEGEIQYLIKWKGWSYIHSTWESEESLQQQKVKGLKKLENFKKKEDEIKQWLG
Mmul_CHD2 ETIEKVLDSRLGKKGATGASTTVYAIEANGDPSGDFDTEKDEGEIQYLIKWKGWSYIHSTWESEESLQQQKVKGLKKLENFKKKEDEIKQWLG
Fcat_CHD2 ETIEKVLDSRLGKKGATGASTTVYAIEANGDPSGDFDTEKDEGEVQYLIKWKGWSYIHSTWESEESLQQQKVKGLKKLENFKKKEDEIKQWLG
Rnor_CHD2 ETIEKVLDSRLGKKGATGASTTVYAVEANGDPSGDFDTEREEGEIQYLIKWKGWSYIHSTWESEESLQQQKVKGLKKLENFKKKEDEIKQWLG
Mmus_CHD2 ETIEKVLDSRLGKKGATGASTTVYAVEANGDPSDDFDTEREEGEVQYLIKWKGWSYIHSTWESEDSLQQQKVKGLKKLENFKKKEDEVKQWLG
```

Chromo 2 (CHD2)
```
Hsap_CHD2 QIVERVIAVKTSKSTLGQTDFPAHSRKPAPSNEPEYLCKWMGLPYSECSWEDEALIGKKFQNCIDSFHSRNNSKTIPTR
Ptro_CHD2 QIVERVIAVKTSKSTLGQTDFPAHSRKPAPSNEPEYLCKWMGLPYSECSWEDEALIGKKFQNCIDSFHSRNNSKTIPTR
Mmul_CHD2 QIVERVIAVKTSKSTLGQTDFPAHSRKPAPSNEPEYLCKWMGLPYSECSWEDEALIGKKFQNCIDSFHSRNNSKTIPTR
Fcat_CHD2 QIVERIIAVKTSKSTLGQSDFPAHSRKPAPSNEPEYLCKWMGLPYSECSWEDEALIGKKFQSCIDSFHSRNNSKTIPTR
Rnor_CHD2 QIVERVIAVKTSKSALGQTDFPAHSRKPAPSNEPEYLCKWMGLPYSECSWEDEALIGKKFQSCIDSFHSRNNSKTIPTR
Mmus_CHD2 QIVERVIAVKTSKSTLGQTDFPAHSRKPAPSNEPEYLCKWMGLPYSECSWEDEALIGKKFQNCIDSFHSRNNSKTIPTR
```

Helicase ATP-Binding (CHD2)
```
Hsap_CHD2 AHSWCKNNSVILADEMGLGKTIQTISFLSYLFHQHQLYGPFLIVVPLSTLTSWQREFEIWAPEINVVVYIGDLMSRNTIREYEWIHSQTKRLK
Ptro_CHD2 AHSWCKNNSVILADEMGLGKTIQTISFLSYLFHQHQLYGPFLIVVPLSTLTSWQREFEIWAPEINVVVYIGDLMSRNTIREYEWIHSQTKRLK
Mmul_CHD2 AHSWCKNNSVILADEMGLGKTIQTISFLSYLFHQHQLYGPFLIVVPLSTLTSWQREFEIWAPEINVVVYIGDLMSRNTIREYEWIHSQTKRLK
Fcat_CHD2 AHSWCKSNSVILADEMGLGKTIQTISFLSYLFHQHQLYGPFLIVVPLSTLTSWQREFEIWAPEINVVVYIGDLMSRNTIREYEWIHSQTKRLK
```

Rnor_CHD2 AHSWCKSNSVILADEMGLGKTIQTISFLSYLFHQHQLYGPFLIVVPLSTLTSWQREFEIWAPEVNVVVYIGDLMSRNTIREYEWIHSQTKRLK
Mmus_CHD2 AHSWCKSNSVILADEMGLGKTIQTISFLSYLFHQHQLYGPFLIVVPLSTLTSWQREFEIWAPEINVVVYIGDLMSRNTIREYEWIHSQTKRLK

Hsap_CHD2 FNALITTYEILLKDKTVLGSINWAFLGVDEAHRLKNDDSLLYKTLIDFKSNHRLLITGTPLQNSLKELWSLLHFIMPE
Ptro_CHD2 FNALITTYEILLKDKTVLGSINWAFLGVDEAHRLKNDDSLLYKTLIDFKSNHRLLITGTPLQNSLKELWSLLHFIMPE
Mmul_CHD2 FNALITTYEILLKDKTVLGSINWAFLGVDEAHRLKNDDSLLYKTLIDFKSNHRLLITGTPLQNSLKELWSLLHFIMPE
Fcat_CHD2 FNALITTYEILLKDKTVLGSINWAFLGVDEAHRLKNDDSLLYKTLIDFKSHHRLLITGTPLQNSLKELWSLLHFIMPE
Rnor_CHD2 FNALITTYEILLKDKTVLGSINWAFLGVDEAHRLKNDDSLLYKTLIDFKSNHRLLITGTPLQNSLKELWSLLHFIMPE
Mmus_CHD2 FNALITTYEILLKDKTVLGSINWAFLGVDEAHRLKNDDSLLYKTLIDFKSNHRLLITGTPLQNSLKELWSLLHFIMPE

Helicase C-Terminal (CHD2)
Hsap_CHD2 LLDKLLTRLRERGNRVLIFSQMVRMLDILAEYLTIKHYPFQRLDGSIKGEIRKQALDHFNADGSEDFCFLLSTRAGGLGINLASADTVVIFDS
Ptro_CHD2 LLDKLLTRLRERGNRVLIFSQMVRMLDILAEYLTIKHYPFQRLDGSIKGEIRKQALDHFNADGSEDFCFLLSTRAGGLGINLASADTVVIFDS
Mmul_CHD2 LLDKLLTRLRERGNRVLIFSQMVRMLDILAEYLTIKHYPFQRLDGSIKGEIRKQALDHFNADGSEDFCFLLSTRAGGLGINLASADTVVIFDS
Fcat_CHD2 LLDKLLTRLRERGNRVLIFSQMVRMLDILAEYLTIKHYPFQRLDGSIKGEIRKQALDHFNADGSEDFCFLLSTRAGGLGINLASADTVVIFDS
Rnor_CHD2 LLDKLLTRLRERGNRVLIFSQMVRMLDILAEYLTIKHYPFQRLDGSIKGEIRKQALDHFNADGSEDFCFLLSTRAGGLGINLASADTVVIFDS
Mmus_CHD2 LLDKLLTRLRERGNRVLIFSQMVRMLDILAEYLTIKHYPFQRLDGSIKGEIRKQALDHFNADGSEDFCFLLSTRAGGLGINLASADTVVIFDS

Hsap_CHD2 DWNPQNDLQAQARAHRIGQKKQVNIYRLVTKGTVEEEIIERAKKKMVLDHLVIQRMDTT
Ptro_CHD2 DWNPQNDLQAQARAHRIGQKKQVNIYRLVTKGTVEEEIIERAKKKMVLDHLVIQRMDTT
Mmul_CHD2 DWNPQNDLQAQARAHRIGQKKQVNIYRLVTKGTVEEEIIERAKKKMVLDHLVIQRMDTT
Fcat_CHD2 DWNPQNDLQAQARAHRIGQKKQVNIYRLVTKGTVEEEIIERAKKKMVLDHLVIQRMDTT
Rnor_CHD2 DWNPQNDLQAQARAHRIGQKKQVNIYRLVTKGTVEEEIIERAKKKMVLDHLVIQRMDTT
Mmus_CHD2 DWNPQNDLQAQARAHRIGQKKQVNIYRLVTKGTVEEEIIERAKKKMVLDHLVIQRMDTT

SANT (CHD2)
CHD2_Hsap GFTDAEIRRFIKAYKKFGLPLERLECIARDAELVDKSVADLKRLGELIHNSCVSAMQEYEEQLKENASEGKGPGKRRGPTIKISGVQVN
CHD2_Ptro GFTDAEIRRFIKAYKKFGLPLERLECIARDAELVDKSVADLKRLGELIHNSCVSAMQEYEEQLKENASEGKGPGKRRGPTIKISGVQVN
CHD2_Mmul GFTDAEIRRFIKAYKKFGLPLERLECIARDAELVDKSVADLKRLGELIHNSCVSAMQEYEEQLKENASEGKGPGKRRGPTIKISGVQVN
CHD2_Fcat GFTDAEIRRFIKAYKKFGLPLERLECIARDAELVDKSVADLKRLGELIHNSCVSAMQEYEEQLKENASEGKGPGKKRGPTIKISGVQVN
CHD2_Rnor GFTDAEIRRFIKAYKKFGLPLERLECIARDAELVDKSVADLKRLGELVHNSCVSAMQEYEEQLKENASEGKGPGKRRGPTIKISGVQVN
CHD2_Mmus GFTDAEIRRFIKAYKKFGLPLERLECIARDAELVDKSVADLKRLGELIHNSCVSAMQEYEEQLKESTSEGKGPGKRRGPTIKISGVQVN

DUF4208 (CHD2)
CHD2_Hsap ICKERMRPVKKALKQLDKPDKGLNVQEQLEHTRNCLLKIGDRIAECLKAYSDQEHIKLWRRNLWIFVSKFTEFDARKLHKLYK
CHD2_Ptro ICKERMRPVKKALKQLDKPDKGLNVQEQLEHTRNCLLKIGDRIAECLKAYSDQEHIKLWRRNLWIFVSKFTEFDARKLHKLYK
CHD2_Mmul ICKERMRPVKKALKQLDKPDKGLNVQEQLEHTRNCLLKIGDRIAECLKAYSDQEHIKLWRRNLWIFVSKFTEFDARKLHKLYK
CHD2_Fcat ICKERMRPVKKALKQLDKPDKGLNVQEQLEHTRNCLLKIGDRIAECLKAYSDQEHIKLWRRNLWIFVSKFTEFDARKLHKLYK
CHD2_Rnor ICKERMRPVKKALKQLDKPDKGLNVQEQLEHTRSCLLKIGDRIAECLRAYSEQEHIKLWRRNLWIFVSKFTEFDARKLHKLYK
CHD2_Mmus ICKERMRPVKKALKQLDKPDKGLSVQEQLEHTRNCLLKIGDRIAECLKAYSDQEHIKLWRRNLWIFVSKFTEFDARKLHKLYK

GROUP II

PHD I (CHD3)
Hsap_CHD3 QDYCEVCQQGGEIILCDTCPRAYHLVCLDPELDRAPEGKWSCPHCEKE
Ptro_CHD3 QDYCEVCQQGGEIILCDTCPRAYHLVCLDPELDRAPEGKWSCPHCEKE
Mmul_CHD3 QDYCEVCQQGGEIILCDTCPRAYHLVCLDPELDRAPEGKWSCPHCEKE
Fcat_CHD3 QDYCEVCQQGGEIILCDTCPRAYHLVCLDPELDRAPEGKWSCPHCEKE
Rnor_CHD3 QDYCEVCQQGGEIILCDTCPRAYHLVCLDPELDRAPEGKWSCPHCEKE
Mmus_CHD3 QDYCEVCQQGGEIILCDTCPRAYHLVCLDPELDRAPEGKWSCPHCEKE

PHD II (CHD3)
Hsap_CHD3 MEYCRVCKDGGELLCCDACISSYHIHCLNPPLPDIPNGEWLCPRCTCP
Ptro_CHD3 MEYCRVCKDGGELLCCDACISSYHIHCLNPPLPDIPNGEWLCPRCTCP
Mmul_CHD3 MEYCRVCKDGGELLCCDACISSYHIHCLNPPLPDIPNGEWLCPRCTCP
Fcat_CHD3 MEYCRVCKDGGELLCCDACISSYHIHCLNPPLPDIPNGEWLCPRCTCP
Rnor_CHD3 MEYCRVCKDGGELLCCDACISSYHIHCLNPPLPDIPNGEWLCPRCTCP
Mmus_CHD3 MEYCRVCKDGGELLCCDACISSYHIHCLNPPLPDIPNGEWLCPRCTCP

Chromo 1 (CHD3)
Hsap_CHD3 EWLCPRCTCPVLKGRVQKILHWRWGEPPVAVPAPQQADGNPDVPPPRPLQGRSEREFFVKWVGLSYWHCSWAKELQLEIFHLVMYRNYQRKNDMDEPPPLD
Ptro_CHD3 EWLCPRCTCPVLKGRVQKILHWRWGEPPVAVPAPQQADGNPDVPPPRPLQGRSEREFFVKWVGLSYWHCSWAKELQLEIFHLVMYRNYQRKNDMDEPPPLD
Mmul_CHD3 EWLCPRCTCPVLKGRVQKILHWRWGEPPVAMPAPQQADGNPDVPAPRPLQGRSEREFFVKWVGLSYWHCSWAKELQLEIFHLVMYRNYQRKNDMDEPPPLD
Fcat_CHD3 EWLCPRCTCPVLKGRVQKILHWRWGEPPVSVPAPQQADGNPDAPPPRPLQGRSEREFFVKWVGLSYWHCSWAKELQLEIFHLVMYRNYQRKNDMDEPPPLD
Rnor_CHD3 EWLCPRCTCPVLKGRVQKILHWRWGEPPVAVPAPQQADGDPDAPPPRVLQGRSEREFFVKWVGLSYWHCSWAKELQLEIFHLVMYRNYQRKNDMDEPPPLD
Mmus_CHD3 EWLCPRCTCPVLKGRVQKILHWRWGEPPVAVPAPQQADGNPDVPPPRPLQGRSEREFFVKWVGLSYWHCSWAKELQLEIFHLVMYRNYQRKNDMDEPPPLD

Chromo 2 (CHD3)
Hsap_CHD3 MTVHRIINHSVDKKGNYHYLVKWRDLPYDQSTWEEDEMNIPEY
Ptro_CHD3 MTVHRIINHSVDKKGNYHYLVKWRDLPYDQSTWEEDEMNIPEY
Mmul_CHD3 MTVHRIINHSVDKKGNYHYLVKWRDLPYDQSTWEEDEMNIPEY
Fcat_CHD3 MTVHRIINHSVDKKGSYHYLVKWRDLPYDQSTWEEDEMSIPEY
Rnor_CHD3 MTVHRIINHSMDKKGNYHYLVKWKDLPYDQSTWEEDEMNIPEY
Mmus_CHD3 MTVHRIINHSMDKKGNYHYLVKWKDLPYDQSTWEEDEMNIPEY

Helicase ATP-Binding (CHD3)
Hsap_CHD3 RFSWAQGTDTILADEMGLGKTIQTIVFLYSLYKEGHTKGPFLVSAPLSTIINWEREFQMWAPKFYVVTYTGDKDSRAIIRENEFSFEDNAIKGGKKAF
Ptro_CHD3 RFSWAQGTDTILADEMGLGKTIQTIVFLYSLYKEGHTKGPFLVSAPLSTIINWEREFQMWAPKFYVVTYTGDKDSRAIIRENEFSFEDNAIKGGKKAF
Mmul_CHD3 RFSWAQGTDTILADEMGLGKTIQTIVFLYSLYKEGHTKGPFLVSAPLSTIINWEREFQMWAPKFYVVTYTGDKDSRAIIRENEFSFEDNAIKGGKKAF
Fcat_CHD3 RFSWAQGTDTILADEMGLGKTIQTIVFLYSLYKEGHTKGPFLVSAPLSTIINWEREFQMWAPKFYVVTYTGDKDSRAIIRENEFSFEDNAIKGGKKAF
Rnor_CHD3 RFSWAQGTDTILADEMGLGKTIQTIVFLYSLYKEGHTKGPFLVSAPLSTIINWEREFQMWAPKFYVVTYTGDKDSRAIIRENEFSFEDNAIKGGKKAF

Mmus_CHD3 RFSWAQGTDTILADEMGLGKTIQTIVFLYSLYKEGHTKGPFLVSAPLSTIINWEREFQMWAPKFYVVTYTGDKDSRAIIRENEFSFEDNAIKGGKKAF

Hsap_CHD3 KMKREAQVKFHVLLTSYELITIDQAALGSIRWACLVVDEAHRLKNNQSKFFRVLNGYKIDHKLLLTGTPLQNNLEELFHLLNFLTPE
Ptro_CHD3 KMKREAQVKFHVLLTSYELITIDQAALGSIRWACLVVDEAHRLKNNQSKFFRVLNGYKIDHKLLLTGTPLQNNLEELFHLLNFLTPE
Mmul_CHD3 KMKREAQVKFHVLLTSYELITIDQAALGSIRWACLVVDEAHRLKNNQSKFFRVLNGYKIDHKLLLTGTPLQNNLEELFHLLNFLTPE
Fcat_CHD3 KMKREAQVKFHVLLTSYELITIDQAALGSIRWACLVVDEAHRLKNNQSKFFRVLNGYKIDHKLLLTGTPLQNNLEELFHLLNFLTPE
Rnor_CHD3 KMKREAQVKFHVLLTSYELITIDQAALGSIRWACLVVDEAHRLKNNQSKFFRVLNGYKIDHKLLLTGTPLQNNLEELFHLLNFLTPE
Mmus_CHD3 KMKREAQVKFHVLLTSYELITIDQAALGSIRWACLVVDEAHRLKNNQSKFFRVLNGYKIDHKLLLTGTPLQNNLEELFHLLNFLTPE

Helicase C-Terminal (CHD3)
Hsap_CHD3 LLQKMLRKLKEQGHRVLIFSQMTKMLDLLEDFLDYEGYKYERIDGGITGALRQEAIDRFNAPGAQQFCFLLSTRAGGLGINLATADTVIIFDSDWNPH
Ptro_CHD3 LLQKMLRKLKEQGHRVLIFSQMTKMLDLLEDFLDYEGYKYERIDGGITGALRQEAIDRFNAPGAQQFCFLLSTRAGGLGINLATADTVIIFDSDWNPH
Mmul_CHD3 LLQKMLRKLKEQGHRVLIFSQMTKMLDLLEDFLDYEGYKYERIDGGITGALRQEAIDRFNAPGAQQFCFLLSTRAGGLGINLATADTVIIFDSDWNPH
Fcat_CHD3 LLQKMLRKLKEQGHRVLIFSQMTKMLDLLEDFLDYEGYKYERIDGGITGALRQEAIDRFNAPGAQQFCFLLSTRAGGLGINLATADTVIIFDSDWNPH
Rnor_CHD3 LLQKMLRKLKEQGHRVLIFSQMTKMLDLLEDFLDYEGYKYERIDGGITGALRQEAIDRFNAPGAQQFCFLLSTRAGGLGINLATADTVIIFDSDWNPH
Mmus_CHD3 LLQKMLRKLKEQGHRVLIFSQMTKMLDLLEDFLDYEGYKYERIDGGITGALRQEAIDRFNAPGAQQFCFLLSTRAGGLGINLATADTVIIFDSDWNPH

Hsap_CHD3 NDIQAFSRAHRIGQANKVMIYRFVTRASVEERITQVAKRKMMLTHLVVRPGLGSKAGSMSKQELDDIL
Ptro_CHD3 NDIQAFSRAHRIGQANKVMIYRFVTRASVEERITQVAKRKMMLTHLVVRPGLGSKAGSMSKQELDDIL
Mmul_CHD3 NDIQAFSRAHRIGQANKVMIYRFVTRASVEERITQVAKRKMMLTHLVVRPGLGSKAGSMSKQELDDIL
Fcat_CHD3 NDIQAFSRAHRIGQANKVMIYRFVTRASVEERITQVAKRKMMLTHLVVRPGLGSKAGSMSKQELDDIL
Rnor_CHD3 NDIQAFSRAHRIGQANKVMIYRFVTRASVEERITQVAKRKMMLTHLVVRPGLGSKAGSMSKQELDDIL
Mmus_CHD3 NDIQAFSRAHRIGQANKVMIYRFVTRASVEERITQVAKRKMMLTHLVVRPGLGSKAGSMSKQELDDIL

DUF1087 (CHD3)
CHD3_Hsap NVDPDYWEKLLRHHYEQQQEDLARNLGKGKRVRKQVNYNDAAQE
CHD3_Ptro NVDPDYWEKLLRHHYEQQQEDLARNLGKGKRVRKQVNYNDAAQE
CHD3_Mmul NVDPDYWEKLLRHHYEQQQEDLARNLGKGKRVRKQVNYNDAAQE
CHD3_Fcat NVDPDYWEKLLRHHYEQQQEDLARNLGKGKRVRKQVNYNDAAQE
CHD3_Rnor NVDPDYWEKLLRHHYEQQQEDLARNLGKGKRVRKQVNYNDAAQE
CHD3_Mmus NVDPDYWEKLLRHHYEQQQEDLARNLGKGKRVRKQVNYNDAAQE

DUF1086 (CHD3)
CHD3_Hsap PEGRRQSKRQLRNEKDKPLPPLLARVGGNIEVLGFNTRQRKAFLNAVMRWGMPPQDAFTTQWLVRDLRGKTEKEFKAYVSLFMRHLCEPGADGSETFADG
CHD3_Ptro PEGRRQSKRQLRNEKDKPLPPLLARVGGNIEVLGFNTRQRKAFLNAVMRWGMPPQDAFTTQWLVRDLRGKTEKEFKAYVSLFMRHLCEPGADGSETFADG
CHD3_Mmul PEGRRQSKRQLRNEKDKPLPPLLARVGGNIEVLGFNTRQRKAFLNAVMRWGMPPQDAFTTQWLVRDLRGKTEKEFKAYVSLFMRHLCEPGADGSETFADG
CHD3_Fcat PEGRRQSKRQLRNEKDKPLPPLLARVGGNIEVLGFNTRQRKAFLNAVMRWGMPPQDAFTTQWLVRDLRGKTEKEFKAYVSLFMRHLCEPGADGSETFADG
CHD3_Rnor PEGRRQSKRQLRNEKDKPLPPLLARVGGNIEVLGFNTRQRKAFLNAVMRWGMPPQDAFTTQWLVRDLRGKTEKEFKAYVSLFMRHLCEPGADGSETFADG
CHD3_Mmus PEGRRQSKRQLRNEKDKPLPPLLARVGGNIEVLGFNTRQRKAFLNAVMRWGMPPQDAFTTQWLVRDLRGKTEKEFKAYVSLFMRHLCEPGADGSETFADG

CHD3_Hsap VPREGLSRQQVLTRIGVMSLVKKKVQEFEHINGRWSMPEL
CHD3_Ptro VPREGLSRQQVLTRIGVMSLVKKKVQEFEHINGRWSMPEL
CHD3_Mmul VPREGLSRQQVLTRIGVMSLVKKKVQEFEHINGRWSMPEL
CHD3_Fcat VPREGLSRQQVLTRIGVMSLVKKKVQEFEHINGRWSMPEL
CHD3_Rnor VPREGLSRQQVLTRIGVMSLVKKKVQEFEHINGRWSMPEL
CHD3_Mmus VPREGLSRQQVLTRIGVMSLVKKKVQEFEHINGRWSMPEL

PHD I (CHD4)
Hsap_CHD4 QDYCEVCQQGGEIILCDTCPRAYHMVCLDPDMEKAPEGKWSCPHCEKE
Ptro_CHD4 QDYCEVCQQGGEIILCDTCPRAYHMVCLDPDMEKAPEGKWSCPHCEKE
Mmul_CHD4 QDYCEVCQQGGEIILCDTCPRAYHMVCLDPDMEKAPEGKWSCPHCEKE
Fcat_CHD4 QDYCEVCQQGGEIILCDTCPRAYHMVCLDPDMEKAPEGKWSCPHCEKE
Rnor_CHD4 QDYCEVCQQGGEIILCDTCPRAYHMVCLDPDMEKAPEGKWSCPHCEKE
Mmus_CHD4 QDYCEVCQQGGEIILCDTCPRAYHMVCLDPDMEKAPEGKWSCPHCEKE

PHD II (CHD4)
Hsap_CHD4 MEFCRVCKDGGELLCCDTCPSSYHIHCLNPPLPEIPNGEWLCPRCTCP
Ptro_CHD4 MEFCRVCKDGGELLCCDTCPSSYHIHCLNPPLPEIPNGEWLCPRCTCP
Mmul_CHD4 MEFCRVCKDGGELLCCDTCPSSYHIHCLNPPLPEIPNGEWLCPRCTCP
Fcat_CHD4 MEFCRVCKDGGELLCCDTCPSSYHIHCLNPPLPEIPNGEWLCPRCTCP
Rnor_CHD4 MEFCRVCKDGGELLCCDTCPSSYHIHCLNPPLPEIPNGEWLCPRCTCP
Mmus_CHD4 MEFCRVCKDGGELLCCDTCPSSYHIHCLNPPLPEIPNGEWLCPRCTCP

Chromo 1 (CHD4)
Hsap_CHD4 TCPALKGKVQKILIWKWGQPPSPTPVPRPPDADPNTPSPKPLEGRPERQFFVKWQGMSYWHCSWVSELQLELHCQVMFRNYQRKNDMDEPPSGDFGGDEEK
Ptro_CHD4 TCPALKGKVQKILIWKWGQPPSPTPVPRPPDADPNTPSPKPLEGRPERQFFVKWQGMSYWHCSWVSELQLELHCQVMFRNYQRKNDMDEPPSGDFGGDEEK
Mmul_CHD4 TCPALKGKVQKILIWKWGQPPSPTPVPRPPDADPNTPSPKPLEGRPERQFFVKWQGMSYWHCSWVSELQLELHCQVMFRNYQRKNDMDEPPSGDFGGDEEK
Fcat_CHD4 TCPALKGKVQKILIWKWGQPPSPTPVPRPPDADPNTPSPKPLEGRPERQFFVKWQGMSYWHCSWVSELQLELHCQVMFRNYQRKNDMDEPPSGDFGGDEEK
Rnor_CHD4 TCPALKGKVQKILIWKWGQPPSPTPVPRPPDADPNTPSPKPLEGRPERQFFVKWQGMSYWHCSWVSELQLELHCQVMFRNYQRKNDMDEPPSGDFGGDEEK
Mmus_CHD4 TCPALKGKVQKILIWKWGQPPSPTPVPRPPDADPNTPSPKPLEGRPERQFFVKWQGMSYWHCSWVSELQLELHCQVMFRNYQRKNDMDEPPSGDFGGDEEK

Chromo 2 (CHD4)
Hsap_CHD4 MMIHRILNHSVDKKGHVHYLIKWRDLPYDQASWESEDVEIQDYDLFKQSYWNHRELMRGEEGRPGKKLKKVKLRKL
Ptro_CHD4 MMIHRILNHSVDKKGHVHYLIKWRDLPYDQASWESEDVEIQDYDLFKQSYWNHRELMRGEEGRPGKKLKKVKLRKL
Mmul_CHD4 MMIHRILNHSVDKKGHVHYLIKWRDLPYDQASWESEDVEIQDYDLFKQSYWNHRELMRGEEGRPGKKLKKVKLRKL
Fcat_CHD4 MMIHRILNHSVDKKGHVHYLIKWRDLPYDQASWESEDVEIQDYDLFKQSYWNHRELMRGEEGRPGKKLKKVKLRKL
Rnor_CHD4 MMIHRILNHSVDKKGHVHYLIKWRDLPYDQASWESEDVEIQDYDLFKQSYWNHRELMRGEEGRPGKKLKKVKLRKL
Mmus_CHD4 MMIHRILNHSVDKKGHVHYLIKWRDLPYDQASWESEDVEIQDYDLFKQSYWNHRELMRGEEGRPGKKLKKVKLRKL

Helicase ATP-Binding (CHD4)
Hsap_CHD4 RFSWAQGTDTILADEMGLGKTVQTAVFLYSLYKEGHSKGPFLVSAPLSTIINWEREFEMWAPDMYVVTYVGDKDSRAIIRENEFSFEDNAIRGGKKASR
Ptro_CHD4 RFSWAQGTDTILADEMGLGKTVQTAVFLYSLYKEGHSKGPFLVSAPLSTIINWEREFEMWAPDMYVVTYVGDKDSRAIIRENEFSFEDNAIRGGKKASR
Mmul_CHD4 RFSWAQGTDTILADEMGLGKTVQTAVFLYSLYKEGHSKGPFLVSAPLSTIINWEREFEMWAPDMYVVTYVGDKDSRAIIRENEFSFEDNAIRGGKKASR
Fcat_CHD4 RFSWAQGTDTILADEMGLGKTVQTAVFLYSLYKEGHSKGPFLVSAPLSTIINWEREFEMWAPDMYVVTYVGDKDSRAIIRENEFSFEDNAIRGGKKASR
Rnor_CHD4 RFSWAQGTDTILADEMGLGKTVQTAVFLYSLYKEGHSKGPFLVSAPLSTIINWEREFEMWAPDMYVVTYVGDKDSRAIIRENEFSFEDNAIRGGKKASR

```
Mmus_CHD4 RFSWAQGTDTILADEMGLGKTVQTAVFLYSLYKEGHSKGPFLVSAPLSTIINWEREFEMWAPDMYVVTYVGDKDSRAIIRENEFSFEDNAIRGGKKASR

Hsap_CHD4 MKKEASVKFHVLLTSYELITIDMAILGSIDWACLIVDEAHRLKNNQSKFFRVLNGYSLQHKLLLTGTPLQNNLEELFHLLNFLTPE
Ptro_CHD4 MKKEASVKFHVLLTSYELITIDMAILGSIDWACLIVDEAHRLKNNQSKFFRVLNGYSLQHKLLLTGTPLQNNLEELFHLLNFLTPE
Mmul_CHD4 MKKEASVKFHVLLTSYELITIDMAILGSIDWACLIVDEAHRLKNNQSKFFRVLNGYSLQHKLLLTGTPLQNNLEELFHLLNFLTPE
Fcat_CHD4 MKKEASVKFHVLLTSYELITIDMAILGSIDWACLIVDEAHRLKNNQSKFFRVLNGYSLQHKLLLTGTPLQNNLEELFHLLNFLTPE
Rnor_CHD4 MKKEASVKFHVLLTSYELITIDMAILGSIDWACLIVDEAHRLKNNQSKFFRVLNGYSLQHKLLLTGTPLQNNLEELFHLLNFLTPE
Mmus_CHD4 MKKEASVKFHVLLTSYELITIDMAILGSIDWACLIVDEAHRLKNNQSKFFRVLNGYSLQHKLLLTGTPLQNNLEELFHLLNFLTPE
```

Helicase C-Terminal (CHD4)

```
Hsap_CHD4 LLQKMLKNLKEGGHRVLIFSQMTKMLDLLEDFLEHEGYKYERIDGGITGNMRQEAIDRFNAPGAQQFCFLLSTRAGGLGINLATADTVIIYDSDWNPH
Ptro_CHD4 LLQKMLKNLKEGGHRVLIFSQMTKMLDLLEDFLEHEGYKYERIDGGITGNMRQEAIDRFNAPGAQQFCFLLSTRAGGLGINLATADTVIIYDSDWNPH
Mmul_CHD4 LLQKMLKNLKEGGHRVLIFSQMTKMLDLLEDFLEHEGYKYERIDGGITGNMRQEAIDRFNAPGAQQFCFLLSTRAGGLGINLATADTVIIYDSDWNPH
Fcat_CHD4 LLQKMLKNLKEGGHRVLIFSQMTKMLDLLEDFLEHEGYKYERIDGGITGNMRQEAIDRFNAPGAQQFCFLLSTRAGGLGINLATADTVIIYDSDWNPH
Rnor_CHD4 LLQKMLKNLKEGGHRVLIFSQMTKMLDLLEDFLEHEGYKYERIDGGITGNMRQEAIDRFNAPGAQQFCFLLSTRAGGLGINLATADTVIIYDSDWNPH
Mmus_CHD4 LLQKMLKNLKEGGHRVLIFSQMTKMLDLLEDFLEHEGYKYERIDGGITGNMRQEAIDRFNAPGAQQFCFLLSTRAGGLGINLATADTVIIYDSDWNPH

Hsap_CHD4 NDIQAFSRAHRIGQNKKVMIYRFVTRASVEERITQVAKKKMMLTHLVVRPGL
Ptro_CHD4 NDIQAFSRAHRIGQNKKVMIYRFVTRASVEERITQVAKKKMMLTHLVVRPGL
Ptro_CHD4 NDIQAFSRAHRIGQNKKVMIYRFVTRASVEERITQVAKKKMMLTHLVVRPGL
Mmul_CHD4 NDIQAFSRAHRIGQNKKVMIYRFVTRASVEERITQVAKKKMMLTHLVVRPGL
Fcat_CHD4 NDIQAFSRAHRIGQNKKVMIYRFVTRASVEERITQVAKKKMMLTHLVVRPGL
Rnor_CHD4 NDIQAFSRAHRIGQNKKVMIYRFVTRASVEERITQVAKKKMMLTHLVVRPGL
Mmus_CHD4 NDIQAFSRAHRIGQNKKVMIYRFVTRASVEERITQVAKKKMMLTHLVVRPGL
```

DUF1087 (CHD4)

```
CHD4_Hsap IIKQEESVDPDYWEKLLRHHYEQQQEDLARNLGKGKRIRKQVNYNDGSQE
CHD4_Ptro IIKQEESVDPDYWEKLLRHHYEQQQEDLARNLGKGKRIRKQVNYNDGSQE
CHD4_Mmul IIKQEESVDPDYWEKLLRHHYEQQQEDLARNLGKGKRIRKQVNYNDGSQE
CHD4_Fcat IIKQEESVDPDYWEKLLRHHYEQQQEDLARNLGKGKRIRKQVNYNDGSQE
CHD4_Rnor IIKQEESVDPDYWEKLLRHHYEQQQEDLARNLGKGKRIRKQVNYNDGSQE
CHD4_Mmus IIKQEESVDPDYWEKLLRHHYEQQQEDLARNLGKGKRIRKQVNYNDGSQE
```

DUF1086 (CHD4)

```
CHD4_Hsap RRPSRKGLRNDKDKPLPPLLARVGGNIEVLGFNARQRKAFLNAIMRYGMPPQDAFTTQWLVRDLRGKSEKEFKAYVSLFMRHLCEPGADGAETFADGV
CHD4_Ptro RRPSRKGLRNDKDKPLPPLLARVGGNIEVLGFNARQRKAFLNAIMRYGMPPQDAFTTQWLVRDLRGKSEKEFKAYVSLFMRHLCEPGADGAETFADGV
CHD4_Mmul RRPSRKGLRNDKDKPLPPLLARVGGNIEVLGFNARQRKAFLNAIMRYGMPPQDAFTTQWLVRDLRGKSEKEFKAYVSLFMRHLCEPGADGAETFADGV
CHD4_Fcat RRPSRKGLRNDKDKPLPPLLARVGGNIEVLGFNARQRKAFLNAIMRYGMPPQDAFTTQWLVRDLRGKSEKEFKAYVSLFMRHLCEPGADGAETFADGV
CHD4_Rnor RRPSRKGLRNDKDKPLPPLLARVGGNIEVLGFNARQRKAFLNAIMRYGMPPQDAFTTQWLVRDLRGKSEKEFKAYVSLFMRHLCEPGADGAETFADGV
CHD4_Mmus RRPSRKGLRNDKDKPLPPLLARVGGNIEVLGFNARQRKAFLNAIMRYGMPPQDAFTTQWLVRDLRGKSEKEFKAYVSLFMRHLCEPGADGAETFADGV

CHD4_Hsap PREGLSRQHVLTRIGVMSLIRKKVQEFEHVNGRWSMPEL
CHD4_Ptro PREGLSRQHVLTRIGVMSLIRKKVQEFEHVNGRWSMPEL
CHD4_Mmul PREGLSRQHVLTRIGVMSLIRKKVQEFEHVNGRWSMPEL
CHD4_Fcat PREGLSRQHVLTRIGVMSLIRKKVQEFEHVNGRWSMPEL
CHD4_Rnor PREGLSRQHVLTRIGVMSLIRKKVQEFEHVNGRWSMPEL
CHD4_Mmus PREGLSRQHVLTRIGVMSLIRKKVQEFEHVNGRWSMPEL
```

PHD I (CHD5)

```
Hsap_CHD5 QDYCEVCQQGGEIILCDTCPRAYHLVCLDPELEKAPEGKWSCPHCEKE
Ptro_CHD5 QDYCEVCQQGGEIILCDTCPRAYHLVCLDPELEKAPEGKWSCPHCEKE
Mmul_CHD5 QDYCEVCQQGGEIILCDTCPRAYHLVCLDPELEKAPEGKWSCPHCEKE
Fcat_CHD5 QDYCEVCQQGGEIILCDTCPRAYHLVCLDPELEKAPEGKWSCPHCEKE
Rnor_CHD5 QDYCEVCQQGGEIILCDTCPRAYHLVCLDPELEKAPEGKWSCPHCEKE
Mmus_CHD5 QDYCEVCQQGGEIILCDTCPRAYHLVCLDPELEKAPEGKWSCPHCEKE
```

PHD II (CHD5)

```
Hsap_CHD5 MEFCRVCKDGGELLCCDACPSSYHLHCLNPPLPEIPNGEWLCPRCTCP
Ptro_CHD5 MEFCRVCKDGGELLCCDACPSSYHLHCLNPPLPEIPNGEWLCPRCTCP
Mmul_CHD5 MEFCRVCKDGGELLCCDACPSSYHLHCLNPPLPEIPNGEWLCPRCTCP
Fcat_CHD5 MEFCRVCKDGGELLCCDACPSSYHLHCLNPPLPEIPNGEWLCPRCTCP
Rnor_CHD5 MEFCRVCKDGGELLCCDACPSSYHLHCLNPPLPEIPNGEWLCPRCTCP
Mmus_CHD5 MEFCRVCKDGGELLCCDACPSSYHLHCLNPPLPEIPNGEWLCPRCTCP
```

Chromo 1 (CHD5)

```
Hsap_CHD5 LPPPKPLEGIPEREFFVKWAGLSYWHCSWVKELQLELYHTVMYRNYQRKNDMDEPPPF
Ptro_CHD5 LPPPKPLEGIPEREFFVKWAGLSYWHCSWVKELQLELYHTVMYRNYQRKNDMDEPPPF
Mmul_CHD5 LPPPKPLEGIPEREFFVKWAGLSYWHCSWVKELQLELYHTVMYRNYQRKNDMDEPPPF
Fcat_CHD5 VPPPKPLEGIPEREFFVKWAGLSYWHCSWVKELQLELYHTVMYRNYQRKNDMDEPPPF
Rnor_CHD5 MPPPRPLEGIPEREFFVKWAGLSYWHCSWVKELQLELYHTVMYRNYQRKNDMDEPPPF
Mmus_CHD5 MPPPRPLEGIPEREFFVKWAGLSYWHCSWVKELQLELYHTVMYRNYQRKNDMDEPPPF
```

Chromo 2 (CHD5)

```
Hsap_CHD5 MMIHRILNHSFDKKGDVHYLIKWKDLPYDQCTWEIDDIDIPYYDNLKQAYWGHRELMLGEDT
Ptro_CHD5 MMIHRILNHSFDKKGDVHYLIKWKDLPYDQCTWEIDDIDIPYYDNLKQAYWGHRELMLGEDT
Mmul_CHD5 MMIHRILNHSFDKKGDVHYLIKWKDLPYDQCTWEIDDIDIPYYDNLKQAYWGHRELMLGEDT
Fcat_CHD5 MMIHRILNHSFDKKGDVHYLIKWKDLPYDQCTWEIDDIDIPYYDNLKQAYWGHRELMLGEDA
Rnor_CHD5 MMVHRILNHSFDKKGDVHYLIKWKDLPYDQCTWEIDEIDIPYYDNLKQTYWGHRELMLGEDA
```

```
Mmus_CHD5 MMVHRILNHSFDKKGDIHYLIKWKDLPYDQCTWEIDEIDIPYYDNLKQAYWGHRELMLGEDA
```

Helicase ATP-Binding (CHD5)

```
Hsap_CHD5 RFSWAQGTDTILADEMGLGKTVQTIVFLYSLYKEGHSKGPYLVSAPLSTIINWEREFEMWAPDFYVVTYTGDKESRSVIRENEFSFEDNAIRSGKKVF
Ptro_CHD5 RFSWAQGTDTILADEMGLGKTVQTIVFLYSLYKEGHSKGPYLVSAPLSTIINWEREFEMWAPDFYVVTYTGDKESRSVIRENEFSFEDNAIRSGKKVF
Mmul_CHD5 RFSWAQGTDTILADEMGLGKTVQTIVFLYSLYKEGHSKGPYLVSAPLSTIINWEREFEMWAPDFYVVTYTGDKESRSVIRENEFSFEDNAIRSGKKVF
Fcat_CHD5 RFSWAQGTDTILADEMGLGKTVQTIVFLYSLYKEGHSKGPYLVSAPLSTIINWEREFEMWAPDFYVVTYTGDKESRSVIRENEFSFEDNAIRSGKKVF
Rnor_CHD5 RFSWAQGTDTILADEMGLGKTVQTIVFLYSLYKEGHSKGPYLVSAPLSTIINWEREFEMWAPDFYVVTYTGDKESRSVIRENEFSFEDNAIRGGKKVF
Mmus_CHD5 RFSWAQGTDTILADEMGLGKTVQTIVFLYSLYKEGHSKGPYLVSAPLSTIINWEREFEMWAPDFYVVTYTGDKESRSVIRENEFSFEDNAIRGGKKVF

Hsap_CHD5 RMKKEVQIKFHVLLTSYELITIDQAILGSIEWACLVVDEAHRLKNNQSKFFRVLNSYKIDYKLLLTGTPLQNNLEELFHLLNFLTPE
Ptro_CHD5 RMKKEVQIKFHVLLTSYELITIDQAILGSIEWACLVVDEAHRLKNNQSKFFRVLNSYKIDYKLLLTGTPLQNNLEELFHLLNFLTPE
Mmul_CHD5 RMKKEVQIKFHVLLTSYELITIDQAILGSIEWACLVVDEAHRLKNNQSKFFRVLNSYKIDYKLLLTGTPLQNNLEELFHLLNFLTPE
Fcat_CHD5 RMKKEVQIKFHVLLTSYELITIDQAILGSIEWACLVVDEAHRLKNNQSKFFRVLNSYKIDYKLLLTGTPLQNNLEELFHLLNFLTPE
Rnor_CHD5 RMKKEVQIKFHVLLTSYELITIDQAILGSIEWACLVVDEAHRLKNNQSKFFRVLNSYKIDYKLLLTGTPLQNNLEELFHLLNFLTPE
Mmus_CHD5 RMKKEVQIKFHVLLTSYELITIDQAILGSIEWACLVVDEAHRLKNNQSKFFRVLNSYKIDYKLLLTGTPLQNNLEELFHLLNFLTPE
```

Helicase C-Terminal (CHD5)

```
Hsap_CHD5 LLQKMLKKLRDEGHRVLIFSQMTKMLDLLEDFLEYEGYKYERIDGGITGGLRQEAIDRFNAPGAQQFCFLLSTRAGGLGINLATADTVIIYDSDWNPHN
Ptro_CHD5 LLQKMLKKLRDEGHRVLIFSQMTKMLDLLEDFLEYEGYKYERIDGGITGGLRQEAIDRFNAPGAQQFCFLLSTRAGGLGINLATADTVIIYDSDWNPHN
Mmul_CHD5 LLQKMLKKLRDEGHRVLIFSQMTKMLDLLEDFLEYEGYKYERIDGGITGGLRQEAIDRFNAPGAQQFCFLLSTRAGGLGINLATADTVIIYDSDWNPHN
Fcat_CHD5 LLQKMLKKLRDEGHRVLIFSQMTKMLDLLEDFLEYEGYKYERIDGGITGGLRQEAIDRFNAPGAQQFCFLLSTRAGGLGINLATADTVIIYDSDWNPHN
Rnor_CHD5 LLQKMLKKLRDEGHRVLIFSQMTKMLDLLEDFLEYEGYKYERIDGGITGGLRQEAIDRFNAPGAQQFCFLLSTRAGGLGINLATADTVIIYDSDWNPHN
Mmus_CHD5 LLQKMLKKLRDEGHRVLIFSQMTKMLDLLEDFLEYEGYKYERIDGGITGGLRQEAIDRFNAPGAQQFCFLLSTRAGGLGINLATADTVIIYDSDWNPHN

Hsap_CHD5 DIQAFSRAHRIGQNKKVMIYRFVTRASVEERITQVAKRKMMLTHLVVRPGLGSKSGSMTKQELDDIL
Ptro_CHD5 DIQAFSRAHRIGQNKKVMIYRFVTRASVEERITQVAKRKMMLTHLVVRPGLGSKSGSMTKQELDDIL
Mmul_CHD5 DIQAFSRAHRIGQNKKVMIYRFVTRASVEERITQVAKRKMMLTHLVVRPGLGSKSGSMTKQELDDIL
Fcat_CHD5 DIQAFSRAHRIGQNKKVMIYRFVTRASVEERITQVAKRKMMLTHLVVRPGLGSKSGSMTKQELDDIL
Rnor_CHD5 DIQAFSRAHRIGQNKKVMIYRFVTRASVEERITQVAKRKMMLTHLVVRPGLGSKSGSMTKQELDDIL
Mmus_CHD5 DIQAFSRAHRIGQNKKVMIYRFVTRASVEERITQVAKRKMMLTHLVVRPGLGSKSGSMTKQELDDIL
```

DUF1087 (CHD5)

```
CHD5_Hsap EDGVEEVEREIIKQEENVDPDYWEKLLRHHYEQQQEDLARNLGKGKRIRKQVNYNDASQE
CHD5_Ptro EDGVEEVEREIIKQEENVDPDYWEKLLRHHYEQQQEDLARNLGKGKRIRKQVNYNDASQE
CHD5_Mmul EDGVEEVEREIIKQEENVDPDYWEKLLRHHYEQQQEDLARNLGKGKRIRKQVNYNDASQE
CHD5_Fcat EDGVEEVEREIIKQEENVDPDYWEKLLRHHYEQQQEDLARNLGKGKRIRKQVNYNDASQE
CHD5_Rnor EDGVEEVEREVIKQEENVDPDYWEKLLRHHYEQQQEDLARNLGKGKRIRKQVNYNDASQE
CHD5_Mmus EDGVEEVEREVIKQEENVDPDYWEKLLRHHYEQQQEDLARNLGKGKRIRKQVNYNDASQE
```

DUF1086 (CHD5)

```
CHD5_Hsap EGQSGRRQSRRQLKSDRDKPLPPLLARVGGNIEVLGFNARQRKAFLNAIMRWGMPPQDAFNSHWLVRDLRGKSEKEFRAYVSLFMRHLCEPGADGAETF
CHD5_Ptro EGQSGRRQSRRQLKSDRDKPLPPLLARVGGNIEVLGFNARQRKAFLNAIMRWGMPPQDAFNSHWLVRDLRGKSEKEFRAYVSLFMRHLCEPGADGAETF
CHD5_Mmul EGQSGRRQSRRQLKSDRDKPLPPLLARVGGNIEVLGFNARQRKAFLNAIMRWGMPPQDAFNSHWLVRDLRGKSEKEFRAYVSLFMRHLCEPGADGAETF
CHD5_Fcat EGQSGRRQSRRQLKSDRDKPLPPLLARVGGNIEVLGFNARQRKAFLNAIMRWGMPPQDAFNSHWLVRDLRGKSEKEFRAYVSLFMRHLCEPGADGAETF
CHD5_Rnor EGQSGRRQSRRQLKSDRDKPLPPLLARVGGNIEVLGFNARQRKAFLNAIMRWGMPPQDAFNSHWLVRDLRGKSEKEFRAYVSLFMRHLCEPGADGAETF
CHD5_Mmus EGQSGRRQSRRQLKSDRDKPLPPLLARVGGNIEVLGFNARQRKAFLNAIMRWGMPPQDAFNSHWLVRDLRGKSEKEFRAYVSLFMRHLCEPGADGAETF

CHD5_Hsap ADGVPREGLSRQHVLTRIGVMSLVRKKVQEFEHVNGKYSTPDL
CHD5_Ptro ADGVPREGLSRQHVLTRIGVMSLVRKKVQEFEHVNGKYSTPDL
CHD5_Mmul ADGVPREGLSRQHVLTRIGVMSLVRKKVQEFEHVNGKYSTPDL
CHD5_Fcat ADGVPREGLSRQHVLTRIGVMSLVRKKVQEFEHVNGKYSTPDL
CHD5_Rnor ADGVPREGLSRQHVLTRIGVMSLVRKKVQEFEHVNGKYSTPDL
CHD5_Mmus ADGVPREGLSRQHVLTRIGVMSLVRKKVQEFEHVNGKYSTPDL
```

Group III

Chromo 1 (CHD6)

```
Hsap_CHD6 NIIEKILASKTVQEVHPGEPPFDLELFYVKYRNFSYLHCKWATMEELEKDPR
Ptro_CHD6 NIIEKILASKTVQEVHPGEPPFDLELFYVKYRNFSYLHCKWATMEELEKDPR
Mmul_CHD6 NIIEKILASKTVQEVHPGEPPFDLELFYVKYRNFSYLHCKWATMEELEKDPR
Fcat_CHD6 NIIEKILASKTVQEVHPGEPPFDLELFYVKYRNFSYLHCKWATMEELEKDPR
Rnor_CHD6 NIIEKILASKTVQEVHPGEPPFDLELFYVKYRNFSYLHCKWATMEELEKDPR
Mmus_CHD6 NIIEKILASKTVQEVHPGEPPFDLELFYVKYRNFSYLHCKWATMEELEKDPR
```

Chromo 2 (CHD6)

```
Hsap_CHD6 VEVDRILEVAHTKDAETGEEVTHYLVKWCSLPYEESTWELEEDVDPAKVKEFESLQVLPEIKHVE
Ptro_CHD6 VEVDRILEVAHTKDAETGEEVTHYLVKWCSLPYEESTWELEEDVDPAKVKEFESLQVLPEIKHVE
Mmul_CHD6 VEVDRILEVAHTKDAETGEEVTHYLVKWCSLPYEESTWELEEDVDPAKVKEFESLQVLPEIKHVE
Fcat_CHD6 VEVDRVLEVAHTKDAETGEEVTHYLVKWCSLPYEESTWELEEDVDPAKVKEFESLQVLPEIKHVE
Rnor_CHD6 IEIDRILEVAHTKDAETGEEVTHYLVKWCSLPYEESTWELEEDVDPAKVKEFESLQILPEVKPVE
Mmus_CHD6 IEIDRILEVAHTKDAETGEEVTHYLVKWCSLPYEESTWELEEDVDPAKVKEFESLQILPEVKHVE
```

Helicase ATP-Binding (CHD6)

```
Hsap_CHD6 LFNWYNRKNCILADEMGLGKTIQSITFLSEIFLRGIHGPFLIIAPLSTITNWEREFRTWTEMNAIVYHGSQISRQMIQQYEMVYRDAQGNP
Ptro_CHD6 LFNWYNRKNCILADEMGLGKTIQSITFLSEIFLRGIHGPFLIIAPLSTITNWEREFRTWTEMNAIVYHGSQISRQMIQQYEMVYRDAQGNP
Mmul_CHD6 LFNWYNRKNCILADEMGLGKTIQSITFLSEIFLRGIHGPFLIIAPLSTITNWEREFRTWTEMNAIVYHGSQISRQMIQQYEMVYRDAQGNP
Fcat_CHD6 LFNWYNRKNCILADEMGLGKTIQSITFLSEIFLRGIHGPFLIIAPLSTITNWEREFRTWTEMNAIVYHGSQISRQMIQQYEMVYRDAQGNP
Rnor_CHD6 LFNWYNRKNCILADEMGLGKTIQSIAFLSEIFVRGIHGPFLIIAPLSTITNWEREFRTWTEMNAIVYHGSQISRQMIQQYEMVYRDAQGNP
```

Mmus_CHD6 LFNWYNRKNCILADEMGLGKTIQSIAFLSEIFVRGIHGPFLIIAPLSTITNWEREFRTWTEMNAIVYHGSQISRQMIQQYEMVYRDAQGNP

Hsap_CHD6 LSGVFKFHVVITTFEMILADCPELKKIHWSCVIIDEAHRLKNRNCKLLEGLKLMALEHKVLLTGTPLQNSVEELFSLLNFLEPS
Ptro_CHD6 LSGVFKFHVVITTFEMILADCPELKKIHWSCVIIDEAHRLKNRNCKLLEGLKLMALEHKVLLTGTPLQNSVEELFSLLNFLEPS
Mmul_CHD6 LSGVFKFHVVITTFEMILADCPELKKIHWSCVIIDEAHRLKNRNCKLLEGLKLMALEHKVLLTGTPLQNSVEELFSLLNFLEPS
Fcat_CHD6 LSGVFKFHVVITTFEMILADCPELKKIHWSCVIIDEAHRLKNRNCKLLEGLKLMALEHKVLLTGTPLQNSVEELFSLLNFLEPS
Rnor_CHD6 LSGVFKFHVVITTFEMILADCPELKKIHWSCVVIDEAHRLKNRNCKLLEGLKLMALEHKVLLTGTPLQNSVEELFSLLNFLEPS
Mmus_CHD6 LSGVFKFHVVITTFEMILADCPELKKIHWSCVIIDEAHRLKNRNCKLLEGLKLMALEHKVLLTGTPLQNSVEELFSLLNFLEPS

Helicase C-Terminal (CHD6)
Hsap_CHD6 LIDKLLPKLIAGGHKVLIFSQMVRCLDILEDYLIQRRYTYERIDGRVRGNLRQAAIDRFCKPDSDRFVFLLCTRAGGLGINLTAADTCIIF
Ptro_CHD6 LIDKLLPKLIAGGHKVLIFSQMVRCLDILEDYLIQRRYTYERIDGRVRGNLRQAAIDRFCKPDSDRFVFLLCTRAGGLGINLTAADTCIIF
Mmul_CHD6 LIDKLLPKLIAGGHKVLIFSQMVRCLDILEDYLIQRRYTYERIDGRVRGNLRQAAIDRFCKPDSDRFVFLLCTRAGGLGINLTAADTCIIF
Fcat_CHD6 LIDKLLPKLIAGGHKVLIFSQMVRCLDILEDYLIQRRYTYERIDGRVRGNLRQAAIDRFCKPDSDRFVFLLCTRAGGLGINLTAADTCIIF
Rnor_CHD6 LIDKLLPKLIAGGHKVLIFSQMVRCLDILEDYLIQRRYTYERIDGRVRGNLRQAAIDRFCKPDSDRFVFLLCTRAGGLGINLTAADTCIIF
Mmus_CHD6 LIDKLLPKLIAGGHKVLIFSQMVRCLDILEDYLIQRRYTYERIDGRVRGNLRQAAIDRFCKPDSDRFVFLLCTRAGGLGINLTAADTCIIF

Hsap_CHD6 DSDWNPQNDLQAQARCHRIGQSKAVKVYRLITRNSYEREMFDKASLKLGLDKAVLQDINRKGGTNGVQQLSKMEVEDLL
Ptro_CHD6 DSDWNPQNDLQAQARCHRIGQSKAVKVYRLITRNSYEREMFDKASLKLGLDKAVLQDINRKGGTNGVQQLSKMEVEDLL
Mmul_CHD6 DSDWNPQNDLQAQARCHRIGQSKAVKVYRLITRNSYEREMFDKASLKLGLDKAVLQDINRKGGTNGVQQLSKMEVEDLL
Fcat_CHD6 DSDWNPQNDLQAQARCHRIGQSKAVKVYRLITRNSYEREMFDKASLKLGLDKAVLQDINRKGSTNGVQQLSKMEVEDLL
Rnor_CHD6 DSDWNPQNDLQAQARCHRIGQSKAVKVYRLITRNSYEREMFDKASLKLGLDKAILQDINRKGSTNGVQQLSKMEVEDLL
Mmus_CHD6 DSDWNPQNDLQAQARCHRIGQSKAVKVYRLITRNSYEREMFDKASLKLGLDKAILQDINRKGSTNGVQQLSKMEVEDLL

SANT (CHD6)
Hsap_CHD6 AQKRWTRREQADFYRTVSSFGVVYDQEKKTFDWTQFRIISRLDKKSDESLEQYFYSFVAM
Ptro_CHD6 AQKRWTRREQADFYRTVSSFGVVYDQEKKTFDWTQFRIISRLDKKSDESLEQYFYSFVAM
Mmul_CHD6 AQKRWTRREQADFYRTVSSFGVVYDQEKKTFDWTQFRIISRLDKKSDESLEQYFYSFVAM
Fcat_CHD6 AQKRWTRREQADFYRAVSSFGVVYDQEKKTFDWTQFRIISRLDKKSDESLEHYFYSFVAM
Rnor_CHD6 AQKRWTRREQADFYRTVSSFGVVYDQEKEAFDWTQFRAISRLDKKSDENLEHYFHSFVAM
Mmus_CHD6 AQKRWTRREQADFYRTVSSFGVVYDQEKKAFDWTQFRIISRLDKKSDESLEHYFYSFVAM

BRK (CHD6)
Hsap_CHD6 SGEERVPAIPKEPGLRGFLPENKFNHTLAEPILRDT
Ptro_CHD6 SGEERVPAIPKEPGLRGFLPENKFNHTLAEPILRDT
Mmul_CHD6 SGEERVPAVPKEPGLRGFLPENKFNHTLGEPVLRDT
Fcat_CHD6 SGEERVPAVPKEPGLRGFLPENKFNHTLAEPVLRDA
Rnor_CHD6 GGEERVSAVPKEPGLRGFLPESKFNHTLAEPVLRDA
Mmus_CHD6 GGEERVPAVPKEPGLRGFLPESKFNHTLAEPVLRDA

Chromo 1 (CHD7)
Hsap_CHD7 PVVEKIMSSRSVKKQKESGEEVEIEEFYVKYKNFSYLHCQWASIEDLEKDKRIQQKIKRFKAKQGQNK
Ptro_CHD7 PVVEKIMSSRSVKKQKESGEEVEIEEFYVKYKNFSYLHCQWASIEDLEKDKRIQQKIKRFKAKQGQNK
Mmul_CHD7 PVVEKIMSSRSVKKQKESGEEVEIEEFYVKYKNFSYLHCQWASVEDLEKDKRIQQKIKRFKAKQGQNK
Fcat_CHD7 PVVEKIMSSRSIKKQKDSGEEIEVEEFYVKYKNFSYLHCQWASVEDLEKDKRIQQKIKRFKAKQGQNK
Rnor_CHD7 PVVEKIMSSRLVKKQKESGEEVEVEEFYVKYKNFSYLHCQWASVEDLEKDKRIQQKIKRFKSKQGQSK
Mmus_CHD7 PVVEKIMSSRLVKKQKESGEEVEVEEFYVKYKNFSYLHCQWASVEDLEKDKRIQQKIKRFKSKQGQSK

Chromo 2 (CHD7)
Hsap_CHD7 VEVDRIMDFARSTDDRGEPVTHYLVKWCSLPYEDSTWERRQDIDQAKIEEFEKLMSREPETERVER
Ptro_CHD7 VEVDRIMDFARSTDDRGEPVTHYLVKWCSLPYEDSTWERRQDIDQAKIEEFEKLMSREPETERVER
Mmul_CHD7 VEVDRIMDFARSTDDRGEPVTHYLVKWCSLPYEDSTWERRQDIDQAKIEEFEKLMSREPETERVER
Fcat_CHD7 VEVDRIMDFARSTDERGEPVTHYLVKWCSLPYEDSTWELRQDIDQAKIEEFEKLMSREPETERVER
Rnor_CHD7 VEVDRIMDFARSTDDRGEPVTHYLVKWCSLPYEDSTWELRQDLDQAKIEEFEKLMSREPETERVER
Mmus_CHD7 VEVDRIMDFARSTDDRGEPVIHYLVKWCSLPYEDSTWELKQDIDQTKIEEFEKLMSREPETERVER

Helicase ATP-Binding (CHD7)
Hsap_CHD7 LFNWYNMRNCILADEMGLGKTIQSITFLYEIYLKGIHGPFLVIAPLSTIPNWEREFRTWTELNVVVYHGSQASRRTIQLYEMYFKDPQGRV
Ptro_CHD7 LFNWYNMRNCILADEMGLGKTIQSITFLYEIYLKGIHGPFLVIAPLSTIPNWEREFRTWTELNVVVYHGSQASRRTIQLYEMYFKDPQGRV
Mmul_CHD7 LFNWYNMRNCILADEMGLGKTIQSITFLYEIYLKGIHGPFLVIAPLSTIPNWEREFRTWTELNVVVYHGSQASRRTIQLYEMYFKDPQGRV
Fcat_CHD7 LFNWYNMRNCILADEMGLGKTIQSITFLYEIYLKGIHGPFLVIAPLSTIPNWEREFRTWTELNVVVYHGSQASRRTIQLYEMYFKDPQGRV
Rnor_CHD7 LFNWYNMRNCILADEMGLGKTIQSITFLYEIYLKGIHGPFLVIAPLSTIPNWEREFRTWTELNVVVYHGSQASRRTIQLYEMYFKDPQGRV
Mmus_CHD7 LFNWYNMRNCILADEMGLGKTIQSITFLYEIYLKGIHGPFLVIAPLSTIPNWEREFRTWTELNVVVYHGSQASRRTIQLYEMYFKDPQGRV

Hsap_CHD7 IKGSYKFHAIITTFEMILTDCPELRNIPWRCVVIDEAHRLKNRNCKLLEGLKMMDLEHKVLLTGTPLQNTVEELFSLLHFLEPS
Ptro_CHD7 IKGSYKFHAIITTFEMILTDCPELRNIPWRCVVIDEAHRLKNRNCKLLEGLKMMDLEHKVLLTGTPLQNTVEELFSLLHFLEPS
Mmul_CHD7 IKGSYKFHAIITTFEMILTDCPELRNIPWRCVVIDEAHRLKNRNCKLLEGLKMMDLEHKVLLTGTPLQNTVEELFSLLHFLEPS
Fcat_CHD7 IKGSYKFHAIITTFEMILTDCPELRNIPWRCVVIDEAHRLKNRNCKLLEGLKMMDLEHKVLLTGTPLQNTVEELFSLLHFLEPS
Rnor_CHD7 IKGSYKFHAIITTFEMILTDCPELRNIPWRCVVIDEAHRLKNRNCKLLEGLKMMDLEHKVLLTGTPLQNTVEELFSLLHFLEPS
Mmus_CHD7 IKGSYKFHAIITTFEMILTDCPELRNIPWRCVVIDEAHRLKNRNCKLLEGLKMMDLEHKVLLTGTPLQNTVEELFSLLHFLEPS

Helicase C-Terminal (CHD7)
Hsap_CHD7 LIDKLLPKLKAGGHRVLIFSQMVRCLDILEDYLIQRRYPYERIDGRVRGNLRQAAIDRFSKPDSDRFVFLLCTRAGGLGINLTAADTCIIF
Ptro_CHD7 LIDKLLPKLKAGGHRVLIFSQMVRCLDILEDYLIQRRYPYERIDGRVRGNLRQAAIDRFSKPDSDRFVFLLCTRAGGLGINLTAADTCIIF
Mmul_CHD7 LIDKLLPKLKAGGHRVLIFSQMVRCLDILEDYLIQRRYPYERIDGRVRGNLRQAAIDRFSKPDSDRFVFLLCTRAGGLGINLTAADTCIIF
Fcat_CHD7 LIDKLLPKLKAGGHRVLIFSQMVRCLDILEDYLIQRRYPYERIDGRVRGNLRQAAIDRFSKPDSDRFVFLLCTRAGGLGINLTAADTCIIF
Rnor_CHD7 LIDKLLPKLKAGGHRVLIFSQMVRCLDILEDYLIQRRYPYERIDGRVRGNLRQAAIDRFSKPDSDRFVFLLCTRAGGLGINLTAADTCIIF

```
Mmus_CHD7 LIDKLLPKLKAGGHRVLIFSQMVRCLDILEDYLIQRRYPYERIDGRVRGNLRQAAIDRFSKPDSDRFVFLLCTRAGGLGINLTAADTCIIF

Hsap_CHD7 DSDWNPQNDLQAQARCHRIGQSKSVKIYRLITRNSYEREMFDKASLKLGLDKAVLQSMSGRENATNGVQQLSKKEIEDLL
Ptro_CHD7 DSDWNPQNDLQAQARCHRIGQSKSVKIYRLITRNSYEREMFDKASLKLGLDKAVLQSMSGRENATNGVQQLSKKEIEDLL
Mmul_CHD7 DSDWNPQNDLQAQARCHRIGQSKSVKIYRLITRNSYEREMFDKASLKLGLDKAVLQSMSGRENATNGVQQLSKKEIEDLL
Fcat_CHD7 DSDWNPQNDLQAQARCHRIGQSKSVKIYRLITRNSYEREMFDKASLKLGLDKAVLQSMSGRENATNGVQQLSKKEIEDLL
Rnor_CHD7 DSDWNPQNDLQAQARCHRIGQSKSVKIYRLITRNSYEREMFDKASLKLGLDKAVLQSMSGRENATNGVQQLSKKEIEDLL
Mmus_CHD7 DSDWNPQNDLQAQARCHRIGQSKSVKIYRLITRNSYEREMFDKASLKLGLDKAVLQSMSGRENATNGVQQLSKKEIEDLL
```

SANT (CHD7)

```
CHD7_Hsap KRQKWTRREEADFYRVVSTFGVIFDPVKQQFDWNQFRAFARLDKKSDESLEKYFSCFVAM
CHD7_Ptro KRQKWTRREEADFYRVVSTFGVIFDPVKQQFDWNQFRAFARLDKKSDESLEKYFSCFVAM
CHD7_Mmul KRQKWTRREEADFYRVVSTFGVIFDPVKQQFDWNQFRAFARLDKKSDESLEKYFSCFVAM
CHD7_Fcat KRQKWTRREEADFYRVVSTFGVIFDPMKQQFDWNQFRAFARLDKKSDESLEKYFSCFVAM
CHD7_Rnor KRQKWTRREEADFYRVVSTFGVIFDPVKQQFDWNQFRAFARLDKKSDESLEKYFSCFVAM
CHD7_Mmus KRQKWTRREEADFYRVVSTFGVIFDPVKQQFDWNQFRAFARLDKKSDESLGKYFSCFVAM
```

BRK (CHD7)

```
Hsap_CHD7 LDPDTRIPVINLEDGTRLVGEDAPKNKDLVEWLKLHPTYTVD
Ptro_CHD7 LDPDTRIPVINLEDGTRLVGEDAPKNKDLVEWLKLHPTYTVD
Mmul_CHD7 LDPDTRIPVINLEDGTRLVGEDAPKNKDLVEWLKLHPTYTVD
Fcat_CHD7 LDPDTRIPVINLEDGTRLVGEDAPKNKDLVEWLKLHPTYTVD
Rnor_CHD7 LDPDTRIPVINLEDGTRLVGEEAPKNKDLVDWLKLHPMYTVD
Mmus_CHD7 LDPDTRIPVINLEDGTRLVGEDAPKNKDLVDWLKLHPTYTVD
```

BRK (CHD7)

```
Hsap_CHD7 TGEERVPVVNKRNGKKMGGAMAPPMKDLPRWLEENPEFAVAPDWTD
Ptro_CHD7 TGEERVPVVNKRNGKKMGGAMAPPMKDLPRWLEENPEFAVAPDWTD
Mmul_CHD7 TGEERVPVVNKRNGKKMGGAMAPPMKDLPRWLEENPEFAVAPDWTD
Fcat_CHD7 TGEERVPVVNKRNGKKMGGAMAPPMKDLPRWLEENPEFAVAPDWTE
Rnor_CHD7 TGEERVPVVNKRNGKKMGGAMAPPMKDLPRWLEENPEFAVAPDWTD
Mmus_CHD7 TGEERVPVVNKRNGKKMGGAMAPPMKDLPRWLEENPEFAVAPDWTD
```

Chromo 1 (CHD8)

```
Hsap_CHD8 AIVDKVLSMRIVKKELPSGQYTEAEEFFVKYKNYSYLHCEWATISQLEKDKRIHQKLKRFKTKMAQMR
Ptro_CHD8 AIVDKVLSMRIVKKELPSGQYTEAEEFFVKYKNYSYLHCEWATISQLEKDKRIHQKLKRFKTKMAQMR
Mmul_CHD8 AIVDKVLSMRIVKKELPSGQYTEAEEFFVKYKNYSYLHCEWATISQLEKDKRIHQKLKRFKTKMAQMR
Fcat_CHD8 AIVDKVLSMRIVKKELPSGQYTEAEEFFVKYKNYSYLHCEWATISQLEKDKRIHQKLKRFKTKMAQMR
Rnor_CHD8 AIVDKVLSMRVVKKELPSGQYTEAEEFFVKYKNYSYLHCEWATISQLEKDKRIHQKLKRFKTKMAQMR
Mmus_CHD8 AIVDKVLSMRVVKKELPSGQYTEAEEFFVKYKNYSYLHCEWATISQLEKDKRIHQKLKRFKTKMAQMR
```

Chromo 2 (CHD8)

```
Hsap_CHD8 VEVDRILDESHSIDKDNGEPVIYYLVKWCSLPYEDSTWELKEDVDEGKIREFKRIQSRHPELKRVNR
Ptro_CHD8 VEVDRILDESHSIDKDNGEPVIYYLVKWCSLPYEDSTWELKEDVDEGKIREFKRIQSRHPELKRVNR
Mmul_CHD8 VEVDRILDESHSIDKDNGEPVIYYLVKWCSLPYEDSTWELKEDVDEGKIREFKRIQSRHPELKRVNR
Fcat_CHD8 VEVDRILDESHSIDKDNGEPVIYYLVKWCSLPYEDSTWELKEDVDEGKIREFKRIQSRHPELKRVNR
Rnor_CHD8 VEVDRILDESHSVDKDNGEPVIYYLVKWCSLPYEDSTWELKEDVDEGKIREFKRIQSRHPELKRVNR
Mmus_CHD8 VEVDRILDESHSVDKDNGEPVIYYLVKWCSLPYEDSTWELKEDVDEGKIREFKRIQSRHPELRRVNR
```

Helicase ATP-Binding (CHD8)

```
Hsap_CHD8 LFNWYNRQNCILADEMGLGKTIQSIAFLQEVYNVGIHGPFLVIAPLSTITNWEREFNTWTEMNTIVYHGSLASRQMIQQYEMYCKDSRGRL
Ptro_CHD8 LFNWYNRQNCILADEMGLGKTIQSIAFLQEVYNVGIHGPFLVIAPLSTITNWEREFNTWTEMNTIVYHGSLASRQMIQQYEMYCKDSRGRL
Mmul_CHD8 LFNWYNRQNCILADEMGLGKTIQSIAFLQEVYNVGIHGPFLVIAPLSTITNWEREFNTWTEMNTIVYHGSLASRQMIQQYEMYCKDSRGRL
Fcat_CHD8 LFNWYNRQNCILADEMGLGKTIQSIAFLQEVYNVGIHGPFLVIAPLSTITNWEREFNTWTEMNTIVYHGSLASRQMIQQYEMYCKDSRGRL
Rnor_CHD8 LFNWYNRQNCILADEMGLGKTIQSIAFLQEVYNVGIHGPFLVIAPLSTITNWEREFNTWTEMNTIVYHGSLASRQMIQQYEMYCKDSRGRL
Mmus_CHD8 LFNWYNRQNCILADEMGLGKTIQSIAFLQEVYNVGIHGPFLVIAPLSTITNWEREFNTWTEMNTIVYHGSLASRQMIQQYEMYCKDSRGRL

Hsap_CHD8 IPGAYKFDALITTFEMILSDCPELREIEWRCVIIDEAHRLKNRNCKLLDSLKHMDLEHKVLLTGTPLQNTVEELFSLLHFLEPS
Ptro_CHD8 IPGAYKFDALITTFEMILSDCPELREIEWRCVIIDEAHRLKNRNCKLLDSLKHMDLEHKVLLTGTPLQNTVEELFSLLHFLEPS
Mmul_CHD8 IPGAYKFDALITTFEMILSDCPELREIEWRCVIIDEAHRLKNRNCKLLDSLKHMDLEHKVLLTGTPLQNTVEELFSLLHFLEPS
Fcat_CHD8 IPGAYKFDALITTFEMILSDCPELREIEWRCVIIDEAHRLKNRNCKLLDSLKHMDLEHKVLLTGTPLQNTVEELFSLLHFLEPS
Rnor_CHD8 IPGAYKFDALITTFEMILSDCPELREIEWRCVIIDEAHRLKNRNCKLLDSLKHMDLEHKVLLTGTPLQNTVEELFSLLHFLEPS
Mmus_CHD8 IPGAYKFDALITTFEMILSDCPELREIEWRCVIIDEAHRLKNRNCKLLDSLKHMDLEHKVLLTGTPLQNTVEELFSLLHFLEPS
```

Helicase C-Terminal (CHD8)

```
Hsap_CHD8 LIDKLLPKLKAGGHKVLIFSQMVRCLDILEDYLIQRRYLYERIDGRVRGNLRQAAIDRFSKPDSDRFVFLLCTRAGGLGINLTAADTCIIF
Ptro_CHD8 LIDKLLPKLKAGGHKVLIFSQMVRCLDILEDYLIQRRYLYERIDGRVRGNLRQAAIDRFSKPDSDRFVFLLCTRAGGLGINLTAADTCIIF
Mmul_CHD8 LIDKLLPKLKAGGHKVLIFSQMVRCLDILEDYLIQRRYLYERIDGRVRGNLRQAAIDRFSKPDSDRFVFLLCTRAGGLGINLTAADTCIIF
Fcat_CHD8 LIDKLLPKLKAGGHKVLIFSQMVRCLDILEDYLIQRRYLYERIDGRVRGNLRQAAIDRFSKPDSDRFVFLLCTRAGGLGINLTAADTCIIF
Rnor_CHD8 LIDKLLPKLKAGGHKVLIFSQMVRCLDILEDYLIQRRYLYERIDGRVRGNLRQAAIDRFSKPDSDRFVFLLCTRAGGLGINLTAADTCIIF
Mmus_CHD8 LIDKLLPKLKAGGHKVLIFSQMVRCLDILEDYLIQRRYLYERIDGRVRGNLRQAAIDRFSKPDSDRFVFLLCTRAGGLGINLTAADTCIIF

Hsap_CHD8 DSDWNPQNDLQAQARCHRIGQSKAVKVYRLITRNSYEREMFDKASLKLGLDKAVLQSMSGR
Ptro_CHD8 DSDWNPQNDLQAQARCHRIGQSKAVKVYRLITRNSYEREMFDKASLKLGLDKAVLQSMSGR
Mmul_CHD8 DSDWNPQNDLQAQARCHRIGQSKAVKVYRLITRNSYEREMFDKASLKLGLDKAVLQSMSGR
Fcat_CHD8 DSDWNPQNDLQAQARCHRIGQSKAVKVYRLITRNSYEREMFDKASLKLGLDKAVLQSMSGR
Rnor_CHD8 DSDWNPQNDLQAQARCHRIGQSKAVKVYRLITRNSYEREMFDKASLKLGLDKAVLQSMSGR
Mmus_CHD8 DSDWNPQNDLQAQARCHRIGQSKAVKVYRLITRNSYEREMFDKASLKLGLDKAVLQSMSGR
```

SANT (CHD8)

```
Hsap_CHD8 KQQRWTRREQTDFYRVVSTFGVEYDPDTMQFHWDRFRTFARLDKKTDESLTKYFHGFVAM
Ptro_CHD8 KQQRWTRREQTDFYRVVSTFGVEYDPDTMQFHWDRFRTFARLDKKTDESLTKYFHGFVAM
Mmul_CHD8 KQQRWTRREQTDFYRVVSTFGVEYDPDTMQFHWDRFRTFARLDKKTDESLTKYFHGFVAM
Fcat_CHD8 KQQRWTRREQTDFYRVVSTFGVEYDPDTMQFHWDRFRTFARLDKKTDESLTKYFHGFVAM
Rnor_CHD8 KQQRWTRREQTDFYRVVSTFGVEYDPDNMQFHWDRFRTFARLDKKTDESLTKYFHGFVAM
```

```
Mmus_CHD8 KQQRWTRREQTDFYRVVSTFGVEYDPDNMQFHWDRFRTFARLDKKTDESLTKYFHGFVAM
```

BRK (CHD8)

```
Hsap_CHD8 DLETRIPVINKVDGTLLVGEDAPRRAELEMWLQGHPEFAVDPRFL
Ptro_CHD8 DLETRIPVINKVDGTLLVGEDAPRRAELEMWLQGHPEFAVDPRFL
Mmul_CHD8 DLETRIPVINKVDGTLLVGEDAPRRAELEMWLQGHPEFAVDPRFL
Fcat_CHD8 DLETRIPVINKVDGTLLVGEDAPRRAELEMWLQGHPEFAVDPRFL
Rnor_CHD8 DLETRIPVINKVDGTLLVGDEAPRRAELDMWLQGHPEFAVDPRFL
Mmus_CHD8 DLETRIPVINKVDGTLLVGDEAPRRAELEMWLQGHPEFAVDPRFL
```

BRK (CHD8)

```
Hsap_CHD8 LGMEPVQTANSRNGKKGHHTETVFNRVLPGPIAPESSKKRA
Ptro_CHD8 LGMEPVQTANSRNGKKGHHTETVFNRVLPGPIAPESSKKRA
Mmul_CHD8 LGMEPVQTANSRNGKKGHHTETVFNRVLPGPIAPESSKKRA
Fcat_CHD8 LGVEPVQTANSRNGKKGHHAETVFNRVLPGPIAPDSSKKRA
Rnor_CHD8 LGMEPVQPANSRNGKKGHYAETAFNRVLPGPIAPENSKKRV
Mmus_CHD8 LGMEPVQPANSRNGKKGHYAETAFNRVLPGPVAPENSKKRV
```

Chromo 1 (CHD9)

```
Hsap_CHD9 AIVDKILSSRTVKKEISPGVMIDTEEFFVKYKNYSYLHCEWATEEQLLKDKRIQQKIKRFKLRQAQRAHFFA
Ptro_CHD9 AIVDKILSSRTVKKEISPGVMIDTEEFFVKYKNYSYLHCEWATEEQLLKDKRIQQKIKRFKLRQAQRAHFFA
Mmul_CHD9 AIVDKILSSRIVKKEISPGVMIDTEEFFVKYKNYSYLHCEWATEEQLLKDKRIQQKIKRFKLRQAQRAHFFA
Fcat_CHD9 AIVDKILSSRITKKEVSPGVMIDTEEFFVKYKNYSYLHCEWATEQQLLKDKRIQQKIKRFKLRQAQRAHFFA
Rnor_CHD9 AIVDKILACRTVKKEVSPGVMLDIEEFFVKYKNYSYLHCEWATEQQLLKDKRIQQKIKRFKLRQAQRAHFLA
Mmus_CHD9 AIVDKILACRTVKKEVSPGVMLDIEEFFVKYKNYSYLHCEWATEQQLLKDKRIQQKIKRFKLRQAQRAHFLA
```

Chromo 2 (CHD9)

```
Hsap_CHD9 VEVDRVLEVSFCEDKDTGEPVIYYLVKWCSLPYEDSTWELKEDVDLAKIEEFEQLQASRPDTRRLDR
Ptro_CHD9 VEVDRVLEVSFCEDKDTGEPVIYYLVKWCSLPYEDSTWELKEDVDLAKIEEFEQLQASRPDTRRLDR
Mmul_CHD9 VEVDRVLEVSFCEDKDTGEPVIYYLVKWCSLPYEDSTWELKEDVDLAKIEEFEQLQASRPDTRRLDR
Fcat_CHD9 VEVDRVLEVSFCEDKDTGEPVIYYLVKWCSLPYEDSTWELKEDVDLAKIEEFEQLQASRPDTRHLDR
Rnor_CHD9 VEVDRILEVSFCEDKDTGESVVYYLVKWCSLPYEDSTWELKEDVDLAKIEEFEQLQASRPDTRQLDR
Mmus_CHD9 VEVDRILEVSFCEDKDTGESVIYYLVKWCSLPYEDSTWELKEDVDLAKIEEFEQLQASRPDTRHLDR
```

Helicase ATP-Binding (CHD9)

```
Hsap_CHD9 LFNWYNRRNCILADEMGLGKTIQSITFLYEILLTGIRGPFLIIAPLSTIANWEREFRTWTDINVVVYHGSLISRQMIQQYEMYFRDSQGRI
Ptro_CHD9 LFNWYNRRNCILADEMGLGKTIQSITFLYEILLTGIRGPFLIIAPLSTIANWEREFRTWTDINVVVYHGSLISRQMIQQYEMYFRDSQGRI
Mmul_CHD9 LFNWYNRRNCILADEMGLGKTIQSITFLYEILLTGIRGPFLIIAPLSTIANWEREFRTWTDINVVVYHGSLISRQMIQQYEMYFRDSQGRI
Fcat_CHD9 LFNWYNRRNCILADEMGLGKTIQSITFLYEILLTGIRGPFLIIAPLSTIANWEREFRTWTDINVVVYHGSLISRQMIQQYEMYFRDSQGRI
Rnor_CHD9 LFNWYNRRNCILADEMGLGKTIQSITFLYEVLLTGIRGPFLIIAPLSTIANWEREFRTWTDINVVVYHGSLISRQMIQQYEMYFRDSQGRI
Mmus_CHD9 LFNWYNRRNCILADEMGLGKTIQSITFLYEILLTGIRGPFLIIAPLSTIANWEREFRTWTDINVVVYHGSLISRQMIQQYEMYFRDSQGRI
```

```
Hsap_CHD9 IRGAYRFQAIITTFEMILGGCGELNAIEWRCVIIDEAHRLKNKNCKLLEGLKLMNLEHKVLLTGTPLQNTVEELFSLLHFLEPL
Ptro_CHD9 IRGAYRFQAIITTFEMILGGCGELNAIEWRCVIIDEAHRLKNKNCKLLEGLKLMNLEHKVLLTGTPLQNTVEELFSLLHFLEPL
Mmul_CHD9 IRGAYRFQAIITTFEMILGGCGELNAIEWRCVIIDEAHRLKNKNCKLLEGLKLMNLEHKVLLTGTPLQNTVEELFSLLHFLEPL
Fcat_CHD9 IRGAYRFQAIITTFEMILGGCGELNAIEWRCVIIDEAHRLKNKNCKLLEGLKLMNLEHKVLLTGTPLQNTVEELFSLLHFLEPL
Rnor_CHD9 IRGAYRFQAIITTFEMILGGCGELNAIEWRCVIIDEAHRLKNKNCKLLEGLKLMNLEHKVLLTGTPLQNTVEELFSLLHFLEPL
Mmus_CHD9 IRGAYRFQAIITTFEMILGGCGELNAIDWRCVIIDEAHRLKNKNCKLLEGLKLMNLEHKVLLTGTPLQNTVEELFSLLHFLEPL
```

Helicase C-Terminal (CHD9)

```
Hsap_CHD9 LIDKLLPKMKAGGHKVLIFSQMVRCLDILEDYLIHKRYLYERIDGRVRGNLRQAAIDRFSKPDSDRFVFLLCTRAGGLGINLTAADTCIIF
Ptro_CHD9 LIDKLLPKMKAGGHKVLIFSQMVRCLDILEDYLIHKRYLYERIDGRVRGNLRQAAIDRFSKPDSDRFVFLLCTRAGGLGINLTAADTCIIF
Mmul_CHD9 LIDKLLPKMKAGGHKVLIFSQMVRCLDILEDYLIHKRYLYERIDGRVRGNLRQAAIDRFSKPDSDRFVFLLCTRAGGLGINLTAADTCIIF
Fcat_CHD9 LIDKLLPKMKAGGHKVLIFSQMVRCLDILEDYLIHKRYLYERIDGRVRGNLRQAAIDRFSKPDSDRFVFLLCTRAGGLGINLTAADTCIIF
Rnor_CHD9 LIDKLLPKMKAGGHKVLIFSQMVRCLDILEDYLIHKRYLYERIDGRVRGNLRQAAIDRFSKPDSDRFVFLLCTRAGGLGINLTAADTCIIF
Mmus_CHD9 LIDKLLPKMKAGGHKVLIFSQMVRCLDILEDYLIHKRYLYERIDGRVRGNLRQAAIDRFSKPDSDRFVFLLCTRAGGLGINLTAADTCIIF
```

```
Hsap_CHD9 DSDWNPQNDLQAQARCHRIGQNKAVKVYRLVTRNSYEREMFDRASLKLGLDKAVLQSMSGR
Ptro_CHD9 DSDWNPQNDLQAQARCHRIGQNKAVKVYRLVTRNSYEREMFDRASLKLGLDKAVLQSMSGR
Mmul_CHD9 DSDWNPQNDLQAQARCHRIGQNKAVKVYRLVTRNSYEREMFDRASLKLGLDKAVLQSMSGR
Fcat_CHD9 DSDWNPQNDLQAQARCHRIGQNKAVKVYRLVTRNSYEREMFDRASLKLGLDKAVLQSMSGR
Rnor_CHD9 DSDWNPQNDLQAQARCHRIGQNKAVKVYRLVTRNSYEREMFDRASLKLGLDKAVLQSMSGR
Mmus_CHD9 DSDWNPQNDLQAQARCHRIGQNKAVKVYRLVTRNSYEREMFDRASLKLGLDKAVLQSMSGR
```

SANT (CHD9)

```
Hsap_CHD9 RQQRWTRREEADFYRVVSTFGVVFDPDRGQFDWTKFRAMARLHKKTDDSLEKYLYAFMSM
Ptro_CHD9 RQQRWTRREEADFYRVVSTFGVVFDPDRGQFDWTKFRAMARLHKKTDDSLEKYLYAFMSM
Mmul_CHD9 RQQRWTRREEADFYRVVSTFGVVFDPDRGQFDWTKFRAMARLHKKTDDSLEKYLYAFMSM
Fcat_CHD9 RQQRWTRREEADFYRVVSTFGVVFDPDRGQFDWTKFRAMARLHKKTDDSLEKYLYAFMSM
Rnor_CHD9 RQQRWTRREEADFYRVVSTFGVVFDPDRGQFDWTKFRALARLHKKTDNSLEKYLCAFMAM
Mmus_CHD9 RQQRWTRREEADFYRVVSTFGVVFDPDRGQFDWTKFRALARLHKKTDNSLEKYLCAFMSM
```

BRK (CHD9)

```
Hsap_CHD9 DTESPVPVINLKDGTRLAGDDAPKRKDLEKWLKEHPGYVEDLGAFIPRMQ
Ptro_CHD9 DTESPVPVINLKDGTRLAGDDAPKRKDLEKWLKEHPGYVEDLGAFIPRMQ
Mmul_CHD9 DTESPVPVINLKDGTRLAGDDAPKRKDLEKWLKEHPGYVEDLGAFIPRMQ
Fcat_CHD9 DTESPVPVINLKDGTRLAGDDAPKRKDLEKWLKEHPGYVEDLGAFIPRMQ
Rnor_CHD9 DTESPVPVINLKDGTRLAGDDAPKRKDLDRWLKEHPGYVEDLGAFIPRVQ
```

```
Mmus_CHD9 DTESPVPVINLKDGTRLAGDDAPKRKDLDRWLKEHPGYVEDLGAFIPRVQ
```

BRK (CHD9)

```
Hsap_CHD9 TGEERVQLINRRNARKVGGAFAPPLKDLCRFLKENSEYGVAPEWG
Ptro_CHD9 TGEERVQLINRRNARKVGGAFAPPLKDLCRFLKENSEYGVAPEWG
Mmul_CHD9 TGEERVQLINRRNARKVGGAFAPPLKDLCRFLKENSEYGVAPEWG
Fcat_CHD9 TGEERVQLINRRNARKVGGAFAPPLKDLCRFLKENSEYGVAPEWG
Rnor_CHD9 TGEERVQLINRRNARKVGGAFAPPLKDLCRFLKENSEYGVAPEWG
Mmus_CHD9 TGEERVQLINRRNARKVGGAFAPPLKDLCRFLKENSEYGVAPEWG
```

Figure S2- Multiple sequence alignment of chromodomains helicase-DNA binding (CHD) protein orthologues (Hsap: *Homo sapiens*; Ptro*: Pan troglodytes*; Mmul: *Macaca mulatta*; Fcat: *Felis catus*; Rnor: *Rattus norvegicus*; Mmus: *Mus musculus*). The grey highlighting represents the variable amino acid positions. The yellow highlighting represents the chromoboxes in Chromo 1 and Chromo 2 and the motifs I, Ia, Ib, Ic, II, III, IV, IVa, V, Va, and VI in Helicase ATP-binding and Helicase C-terminal domains. Bold red residues represent pathogenic mutations retrieved from Clinvar [63] and also described in literature (Table 1).
